# Validation of Reference Genes for RT–qPCR Analysis in Noise–Induced Hearing Loss: A Study in Wistar Rat

**DOI:** 10.1371/journal.pone.0138027

**Published:** 2015-09-14

**Authors:** Pedro Melgar–Rojas, Juan Carlos Alvarado, Verónica Fuentes–Santamaría, María Cruz Gabaldón–Ull, José M. Juiz

**Affiliations:** Instituto de Investigación en Discapacidades Neurológicas (IDINE), School of Medicine, University of Castilla–La Mancha, Campus in Albacete, Albacete, Spain; University of South Florida, UNITED STATES

## Abstract

The reverse transcriptase–quantitative polymerase chain reaction (RT–qPCR) requires adequate normalization in order to ensure accurate results. The use of reference genes is the most common method to normalize RT–qPCR assays; however, many studies have reported that the expression of frequently used reference genes is more variable than expected, depending on experimental conditions. Consequently, proper validation of the stability of reference genes is an essential step when performing new gene expression studies. Despite the fact that RT–qPCR has been widely used to elucidate molecular correlates of noise–induced hearing loss (NIHL), up to date there are no reports demonstrating validation of reference genes for the evaluation of changes in gene expression after NIHL. Therefore, in this study we evaluated the expression of some commonly used reference genes (*Arbp*, *b–Act*, *b2m*, *CyA*, *Gapdh*, *Hprt1*, *Tbp*, *Tfrc* and *UbC*) and examined their suitability as endogenous control genes for RT–qPCR analysis in the adult Wistar rat in response to NIHL. Four groups of rats were noise–exposed to generate permanent cochlear damage. Cochleae were collected at different time points after noise exposure and the expression level of candidate reference genes was evaluated by RT–qPCR using geNorm, NormFinder and BestKeeper software to determine expression stability. The three independent applications revealed *Tbp* as the most stably expressed reference gene. We also suggest a group of top–ranked reference genes that can be combined to obtain suitable reference gene pairs for the evaluation of the effects of noise on gene expression in the cochlea. These findings provide essential basis for further RT–qPCR analysis in studies of NIHL using Wistar rats as animal model.

## Introduction

Noise–induced hearing loss (NIHL) is the main cause of preventable acquired hearing loss among people between 20–69 years [1]. Cochlear damage produced by noise exposure is generated by direct mechanical stress initiated immediately after the exposure which leads to secondary metabolic alterations that progressively induce cell death along several weeks following the lesion [2]. Several mechanisms have been proposed to account for this cellular damage including excessive generation of free radicals following noise exposure, which has been postulated to be involved in the pathogenesis of NIHL [3]. In this regard, previous studies have demonstrated that different free radicals, notably reactive oxygen species (ROS) are generated in the cochlea soon after noise exposure, reaching maximum levels 7–10 days after the insult [4–7]. As a consequence, cells in the cochlea trigger their antioxidant defensive systems against noise–induced oxidative damage [8–12]. Studies have revealed a causal relationship between oxidative stress blocking by antioxidant administration and improvement in hearing [13].

A great effort has been made to elucidate the molecular mechanisms involved in the oxidative stress processes that lead to cell death in the cochlea, which have been described to take place along several weeks after noise exposure [14–16]. There is a correlation between NIHL and outer hair cell (OHC) loss in rats [17]. OHCs die through apoptosis and necrosis, which occur simultaneously in the cochlea after noise exposure [18–24]. In this regard, several studies have evaluated the expression patterns of different key apoptosis regulatory genes in noised–exposed animals at different time points post–exposure [25–39].

One of the most powerful techniques to quantify mRNA levels of different target genes is RT–qPCR [40]. However, RT–qPCR experiments require normalization to compensate and control confounding variability sources along the whole experimental protocol, from RNA extraction to RT–qPCR data analysis [40,41]. The use of reference genes as internal controls is the most common method for normalizing RT–qPCR experiments [41,42]. The ideal reference gene should be stably expressed in the tissue of interest, that is, its expression level should not change along time or under any experimental conditions. However, studies have demonstrated that commonly used reference genes are indeed differentially regulated in different cell types [43]. Thus, the identification of suitable reference genes for RT–qPCR data normalization remains an essential step that should be determined for each new experimental design in order to obtain accurate results.

In the present study, we searched nine candidate reference genes (*Arbp*, *b–Act*, *b2m*, *CyA*, *Gapdh*, *Hprt1*, *Tbp*, *Tfrc* and *UbC*) belonging to several different functional groups and therefore, unlikely to be co–regulated [44]. Some of them have been previously used to evaluate the impact of aged–related hearing loss (ARHL) in the cochlea of Fischer 344 rats [45]. However, considering that the Wistar rat is a well–known model for NIHL [37,38,46–55] and ARHL [56], it is surprising that to date, there are no systematic studies to validate and determine the suitability of those reference genes as internal control genes for RT–qPCR analysis in NIHL experiments.

In this study Wistar rats were noise–exposed to generate permanent auditory damage (permanent threshold shift or PTS) and sacrificed at different time points. At each time point the expression level of each candidate reference gene was evaluated by RT–qPCR. In order to determine the expression stability of reference genes, three most commonly used software programs were employed: geNorm [57], NormFinder [58] and BestKeeper [59]. All of them revealed that regardless of noise overstimulation, *Tbp* was the most stable reference gene at all time points evaluated. We also report different combinations of some top–ranked candidate reference genes (*Tbp*, *Arbp*, *Hprt1* and *b2m*) as optimal options to be used as endogenous control in further studies evaluating the molecular mechanisms involving NIHL in Wistar rats.

## Materials and Methods

### Animals

Twenty–three Wistar rats (female, three–months old) were purchased from Charles River (Barcelona, Spain) and maintained at the Universidad de Castilla–La Mancha Animal house (Albacete, Spain). Upon arrival, animals were housed under controlled conditions (temperature 22–23°C and humidity 60±5%), a 12h light/dark cycle and food/water *ad libitum*. Animal handling and well–being conformed to current national (Spain R.D. 53/2013; Law 32/2007) and EU (Directive 2010/63/EU) regulations regarding protection and care of animals used for scientific purposes. All procedures were approved by the Committee on Ethics of Animal Experimentation of the Universidad de Castilla–La Mancha (Permit Number: PR–2013–02–03).

### Auditory brainstem response (ABR) recordings

ABR recordings were performed, as described elsewhere [60], in a sound–attenuating, electrically shielded booth (EYMASA/INCOTRON S.L., Barcelona, Spain) located inside a sound–attenuating room. Rats were anesthetized with isoflurane (1 L/min O2 flow rate) at 4% for induction and 1.5–2% for maintenance. During recordings, the temperature was monitored with a rectal probe and maintained at 37.5 ± 1°C using a non–electrical heating pad. Subdermal needle electrodes (Rochester Electro–Medical, Tampa, FL, USA) were placed at the vertex (non–inverting) and in the right (inverting) and the left (ground) mastoids. Sound stimulation and recordings were performed using a BioSig System III (Tucker−Davis Technologies, Alachua, FL, USA). The stimuli, generated digitally by the SigGenRP software (Tucker−Davis Technologies) and the RX6 Piranha Multifunction Processor hardware (Tucker−Davis Technologies), consisted of tone bursts (5 ms rise/fall time without a plateau with a cos2 envelope delivered at 20/s) at 7 different frequencies (0.5, 1, 2, 4, 8, 16, and 32 kHz). Then, the stimuli were delivered into the external auditory meatus of the right ear using an EDC1 electrostatic speaker driver (Tucker–Davis Technologies) through an EC−1 electrostatic speaker (Tucker−Davis Technologies). Stimuli were calibrated prior to the experiments using SigCal software (Tucker−Davis Technologies) and an ER−10B+ low noise microphone system (Etymotic Research Inc., Elk, Groove, IL, USA). Evoked responses were filtered (0.3–3.0 kHz), averaged (500 waveforms) and stored for offline analysis. In order to evaluate the auditory threshold, the background activity was measured before the stimulus onset and the evoked responses were recorded in 5 dB steps descending from 80 dB sound pressure level (SPL). The stimulus intensity that evoked waveforms with a peak–to–peak voltage greater than 2 standard deviations (SD) of the background activity was set as the auditory threshold [60–62]. The maximum level of intensity was set at 80 dB to avoid noise overstimulation in control animals and also additional hearing loss in experimental rats [63]. Following noise exposure, if no evoked responses were obtained during the recording at 80 dB, auditory thresholds were set to that value for statistical analysis [64,65].

### Noise exposure and study design

**Fig 1:**
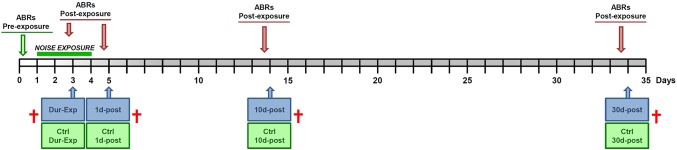
Experimental design. Wistar rats were exposed to broadband noise (0.5–32 kHz, 118 dB SPL), for 4h/day during 4 consecutive days to induce permanent auditory damage. Auditory brainstem responses (ABR) were evaluated prior to exposure and prior to each time point to analyze: during exposure (Dur–Exp) and at 1 day (1d–post), 10 days (10d–post) and 30 days (30d–post) post–exposure. At each time point, cochleae from noise–exposed animals and their matched control were micro–dissected in order to study the stability of candidate reference genes by RT–qPCR.

Animals with positive Preyer’s reflex and normal ABRs were randomly assigned to one of the following groups: Control (Ctrl; n = 7), during exposure (Dur−Exp; see below n = 4), 1 day post–exposure (1d−post; n = 4), 10 days post–exposure (10d−post; n = 4) and 30 days post–exposure (30d−post; n = 4). At day 0, using a double wall sound–attenuating booth located inside a sound–attenuating room, experimental rats were exposed to broadband noise (0.5–32 kHz) at 118 dB SPL, for 4 hours a day during, either 2 consecutive days (assigned to the Dur−Exp group) or 4 consecutive days (assigned to 1d–post, 10d–post or 30d–post groups) (Fig 1). ABRs were performed prior to noise exposure and at each time point post–exposure (Fig 1). After the ABR recordings the animals from experimental and Ctrl groups were euthanized and cochlear tissue processed as described below (Fig 1).

### Cochlear histology

#### Nissl staining

At the corresponding time points after noise exposure, control (n = 3) and experimental (n = 3) rats were anesthetized with an intraperitoneal injection of ketamine (100mg/kg) and xylazine (5mg/kg) and perfused transcardially with 0.9% saline wash followed by a fixative solution of 4% paraformadehyde in 0.1 M phosphate buffer (PB, pH 7.3). Cochleae were quickly removed from the temporal bone and decalcified in 50% RDO rapid decalcifier solution (Apex Engineering Products Corporation, Illinois, USA) for 2h. Following decalcification and after several rinses in PB = 0.1M, the left cochleae from each animal were cryoprotected in a solution of 30% sucrose overnight, embedded in a solution of 15% sucrose and 10% gelatin and frozen at −70°C by immersion in 2–propanol/dry ice bath. The next day, they were sectioned at 20μm on a cryostat, mounted onto SuperFrost slides, Nissl–stained and coverslipped using Cytoseal (Stephens Scientific). Cochlear sections were examined with brightfield illumination using a Nikon Eclipse photomicroscope and images captured with a DXM 1200C digital camera attached to the microscope.

#### Surface preparations of the Organ of Corti

The right cochleae from each animal were processed for whole mount surface preparations. The sensory epitheliums were isolated, and microdissected into individual turns that were mounted on glass slides and stained with DAPI nuclear staining. Cochlear tissue was examined with a laser scanning confocal microscope (LSM 710; Zeiss, Germany) and the images were captured with a 40X objective.

### Cochlear dissection

At the corresponding time points described previously (Fig 1), animals were deeply anesthetized with 1.5–2% isoflurane (1 L/min O_2_ flow rate; Esteve) followed by an intraperitoneal injection of ketamine (80mg/kg; Pfizer) and xylazine (10mg/kg; Laboratorios Calier S.A.). After euthanasia, temporal bones were rapidly removed and placed in cold 1X Phosphate Buffer Saline (PBS). Whole cochleae were isolated using a dissection microscope, collected into cryotubes (Corning, Cat. No. 430489) and immediately frozen on dry ice. The whole process was carried out within 6–8min. Samples were stored at −80°C. All dissection tools were cleaned and treated with RNaseZap (Sigma–Aldrich, Cat. No. R2020) before each dissection.

### RNA extraction and cDNA synthesis

As both cochleae of each animal underwent similar noise–induced damage, only the RNA from one randomly chosen cochlea was extracted. Frozen cochleae were weighed and the corresponding volume of cold TRIzol reagent (Life Technologies, Cat. No. 15596018) was added according to manufacturer’s instructions. Cochleae were then quickly homogenized using a Polytron PT 2100 homogenizer (Kinematica, Dispersing aggregate PT−DA 2105/2EC; Rotor–Ø 3mm) at 30x1000 rpm for < 30 seconds. The Polytron rotor was previously cleaned, treated with RNaseZap (Sigma–Aldrich) and cooled in dry ice for ~1 min. Total RNA was extracted according to TRIzol manufacturer’s instructions. Quantity and quality of RNAs were assessed by electrophotometric (Nanodrop ND−1000, Thermo Scientific) and electrophoretic analysis (0.8% agarose gels; 60V), according to the MIQE guidelines for qPCR ([42], see below). All RNA samples showed suitable A260/A280 and 28S/18S ratios. RNAs were stored at −80°C.

RevertAid First Strand cDNA Synthesis Kit (Thermo Scientific, Cat. No. #K1622) was used to synthesize first–strand cDNAs from 1μg of RNA using oligo–(dT)_18_ as primer. The reactions were performed in a DNA Engine^®^ Peltier Thermal Cycler (BioRad) and, according to manufacturer’s instructions, in a final volume of 20 μl. Reaction conditions were as follows: 65°C for 5 min, 37°C for 5 min, 42°C for 1h, 72°C for 10 min, 4°C for ∞. After the reaction, cDNAs were diluted 1:10 for use in RT−qPCR and stored at 4°C for immediate use or at −20°C for long–term storage. All the RT−qPCR experiments were performed with the same batch of cDNAs. Non reverse transcriptase (–RT) controls were performed in order to determine genomic DNA contamination of the RNA samples by using the *UbC* gene primers. These were the only ones that amplified the same region in both the cDNA and the genomic DNA (see below). The average difference among the *UbC* expression in each sample and the corresponding–RT expression was 14.25 ± 1.09 Cq indicating that no genomic DNA was present in the RNA samples.

### Primer design

RT–qPCR were performed using specific primer pairs for amplifying transcripts of nine reference genes researched from the recent literature (Table 1). Careful attention was paid to select those genes from different functional groups. Theoretically this should reduce the chance that they might be co–regulated [44]. Primer pairs were designed using the specific software Primer3 Plus (available at: http://www.bioinformatics.nl/cgi-bin/primer3plus/primer3plus.cgi/) or selected from the literature ([66–68]; Table 2). Gene specificities were tested by BLAST analysis (NCBI). Moreover, primer pairs were matched against the genomic sequence (downloaded from Ensembl Data Base) to check if they spanned at least two exons or had a large intron between them to avoid false–positive amplification in the case of genomic DNA contamination (Table 2). Amplification efficiencies (E values) and correlation coefficients (R^2^ values) of the reference genes were obtained from the slope of the standard curves (Table 2) as indicated in the MIQE guidelines ([42], see below). Five 10–fold serial dilutions of a control cochlea cDNA were used to calculate the standard curves. Only Cq values less than 35 were used to obtain E values and R^2^ value.

**Table 1:** Reference genes evaluated in this study.

Symbol	Gene name	Function	Localization
*Arbp / Rplp0*	Ribosomal protein, large, P0	Member of the ribosomal protein family.	12q16
*b–Act*	Actin, beta	Structural protein of the cytoskeleton involved in cell motility processes.	12p11
*b2m*	Beta–2 microglobulin	Beta–chain of major histocompatibility complex class I molecules.	3q35
*CyA / Ppia*	Cyclophilin A–Peptidylprolyl isomerase A	Accelerates the protein folding by its activity peptidyl–prolyl cis–trans isomerase.	14q21
*Gapdh*	Glyceraldehyde–3–phosphate dehydrogenase	Glycolytic enzyme that catalyzes a reversible oxidative phosphorylation in glycolysis and gluconeogenesis.	4q42
*Hprt1*	Hypoxanthine phosphoribosyltransferase 1	Catalyzes a central reaction in the synthesis of purine nucleotides.	Xq36
*Tbp*	TATA box binding protein	RNA polymerase II transcription factor that attaches specific DNA sequences known as the TATA box.	1q12
*Tfrc*	Transferrin receptor	Carrier protein for transferrin, needed for the cellular uptake of iron.	11q22
*UbC*	Ubiquitin C	Involved in protein degradation pathways.	12q14

**Table 2:** Oligonucleotides and qPCR parameters.

Gene	GeneBank Accession Number.	Primer sequence (5’–3’)	Genomic location (exons; FW–RV)	Product size (bp)	PCR efficiency	Regression coefficient (R^2^)	Reference
*Arbp / Rplp0*	NM_022402.2	FW: CCCTTCTCCTTCGGGCTGAT; RV: TGAGGCAACAGTCGGGTAGC	4–5	165	91.4%	1.000	[66]
*b–Act*	NM_031144.3	FW: CGCGAGTACAACCTTCTTGC; RV: CGCGAGTACAACCTTCTTGC	1–3	211	92.6%	1.000	This report
*b2m*	NM_012512.2	FW: GTGTCTCAGTTCCACCCACC; RV: TTACATGTCTCGGTCCCAGG	2–2/3^a^	222	104.2%	0.9998	This resport
*CyA / Ppia*	NM_017101.1	FW: ATGGTCAACCCCACCGTGTT; RV: CGTGTGAAGTCACCACCCT	1–3/4	206	92.5%	0.9979	[67]
*Gapdh*	NM_017008.4	FW: AGACAGCCGCATCTTCTTGT; RV: CTTGCCGTGGGTAGAGTCAT	1–3	207	90.9%	0.9975	This report
*Hprt1*	NM_012583.2	FW: TCCCAGCGTCGTGATTAGTGA; RV: CCTTCATGACATCTCGAGCAAG	1/2–3	152	97.3%	0.9996	[66]
*Tbp*	NM_001004198.1	FW: CCCACATCACTGTTTCATGG; RV: CCGTAAGGCATCATTGGACT	1/2–3	215	99.2%	0.9995	This report
*Tfrc*	NM_022712.1	FW: ATCATCAAGCAGCTGAGCCAG; RV: CTCGCCAGACTTTGCTGAATTT	4/5–5	124	94.4%	0.9998	[66]
*UbC*	NM_017314.1	FW: CACCAAGAAGGTCAAACAGGA; RV: GACACCTCCCCATCAAACCC	3–3	101	94.1%	0.9999	This report
*Bad*	NM_022698.1	FW: CAGGCAGCCAATAACAGT; RV: CCATCCCTTCATCTTCCTC	2–3	100	92.7%	0.9951	[68]
*Sod2*	NM_017051.2	FW: CTGGACAAACCTGAGCCCTA; RV: GACCCAAAGTCACGCTTGATA	3–4	77	92.4%	0.9935	This report

^a^Primers that match on an exon–exon junction.

### RT–qPCR

RT–qPCRs were performed in a One Step Plus Real–Time PCR System machine (Applied Biosystems) using 96–well plates and Fast SYBR Green Master Mix (Applied Biosystems, Cat. No. 4385612) as reagent. Briefly, the RT–qPCR reaction mix per well consisted of 2.8μl of sterile H_2_O MilliQ, 0.1μl of each primer (final concentration of 100nM), 5μl of Fast SYBR Green Master Mix and 2μl of 1:10–diluted cDNA. After the reaction mix was dispensed in the corresponding wells, the plate was centrifuged at 1200 rpm for 2 min. The RT–qPCR amplification was performed starting with an initial activation step (95°C for 20 s) followed by 40 cycles of 95°C for 6 s and 60°C for 45 s. The melting curve was generated by an initial denaturation step (95°C for 20 s) followed by a gradual heating from 60°C to 95°C (ramp of 0.3°C).

The melting curve analysis confirmed that the primers amplified only one specific PCR product (S1 Fig). The amplification efficiencies were calculated as: Efficiency (%) = (−1+10^(−1/slope)^) x 100. All the RT–qPCR plates included non–template controls (NTC) which generated Cq values >35. The experiments were performed technically in triplicate and biologically in quadruplicate with the exception of the Ctrl group that contained seven biological samples (see above).

Quantification of expression (fold change) from the Cq data was calculate using Step One Software v2.3 (Applied Biosystems) and following the ∆∆Cq method [69]. Briefly, the expression level of a target gene was first normalized to the average level (the geometric mean as recommended in [57]) of the corresponding reference gene or reference gene pair to obtain the ∆Cq value of each gene pair in the samples (control and noise–exposed). Then, the ∆∆Cq of each gene was calculated as: ∆Cq (noise–exposed group)– ∆Cq (control group), where “noise–exposed group” corresponds to each experimental group: Dur−Exp, 1d−post, 10d−post or 30d−post. To calculate the relative expression (fold change) the following formula was used: 2^–∆∆Cq^.

All RT–qPCR experiments were compliant with the MIQE guidelines ([42], see below).

### Expression stability analysis

The expression stability of the candidate reference genes was examined by three different software algorithms developed for Microsoft Excel: geNorm [57], NormFinder [58] and BestKeeper [59]. For geNorm and Normfinder, the RT–qPCR Cq values were transformed into relative quantities (Q) by the ∆Cq method: Q = (E)^∆Cq^, where E = amplification efficiency of each amplicon and ∆Cq = lowest Cq value–sample Cq value. For BestKeeper, Cq values and E values were the input data.

#### geNorm analysis

The geNorm algorithm is based on the principle that the expression ratio of two appropriate reference genes should be unaffected by the experimental conditions [57]. Then, it firstly calculates the pairwise variation of each candidate reference gene with all other tested genes in order to subsequently determine the stability value (M) for each reference gene as the average of this pairwise variation. The gene showing the lowest M value would be the most stably expressed. On the other hand, geNorm calculates the minimal number of reference genes for accurate normalization from the pairwise variation between two sequential normalization factors containing an increasing number of genes (pairwise variation, V). It proposes a 0.15 cut–off value in such a way that a Vn/n+1 pairwise variation below 0.15 indicates that n genes are sufficient for normalization and the gene n+1 should not be included.

#### NormFinder analysis

The NormFinder software is a Visual Basic Application (VBA) for Excel. In contrast to the other two algorithms, NormFinder takes into account the intra–and inter–group variations [58]. It analyzes the intra–and inter–group variances and calculates a stability value (M) that recapitulates this information. The gene with lowest M value is most stably expressed. It would match with that containing inter–group variance as close to zero as possible and at the same time having the average intra–group variances as small as possible. Normfinder also suggests the best combination of two reference genes and calculates the stability value for this combination.

#### BestKeeper analysis

BestKeeper is based on the principle that proper reference genes should display similar expression patterns and should be highly correlated. This Excel based software calculates several key data for each candidate reference gene from raw Cq values: (1) the coefficient of variation (CV); (2) the standard deviation (SD) of the Cq values; and (3) the coefficient of correlation (r). BestKeeper doesn’t indicate which of these values is the most relevant. CV and SD values give a first estimation of the reference gene stability [59]. In this way, the most stably expressed genes would exhibit lowest variation (lowest CV and lowest SD). Genes with SD > 1 are considered inconsistent. On the other hand, highly correlated genes (with high r values) would be putatively stably expressed [44].

### MIQE Guidelines

In an effort to provide greater transparency and better reproducibility of our results between research laboratories, this study was carried out in compliance to the Minimum Information for Publication of Quantitative Real–Time PCR Experiments (MIQE; [42]). In this regard, the MIQE checklist is provided (S1 Table).

### Statistical analysis

For statistical analysis GraphPad Prism 5.0 software (GraphPad Software Inc) for Windows was used. Depending on whether the sample distributions met the assumptions of normality based on the Kolmogorov–Smirnov test of normality, Student’s t test was used to compare different groups of data. When the assumption was not met, the Mann–Whitney test was used. All data are expressed as means ± SD (S2 and S3 Tables). Statistical significance was defined as *p<0.05, **p<0.01, ***p<0.001 (S4 and S5 Tables).

## Results

### Auditory Brainstem Responses

**Fig 2:**
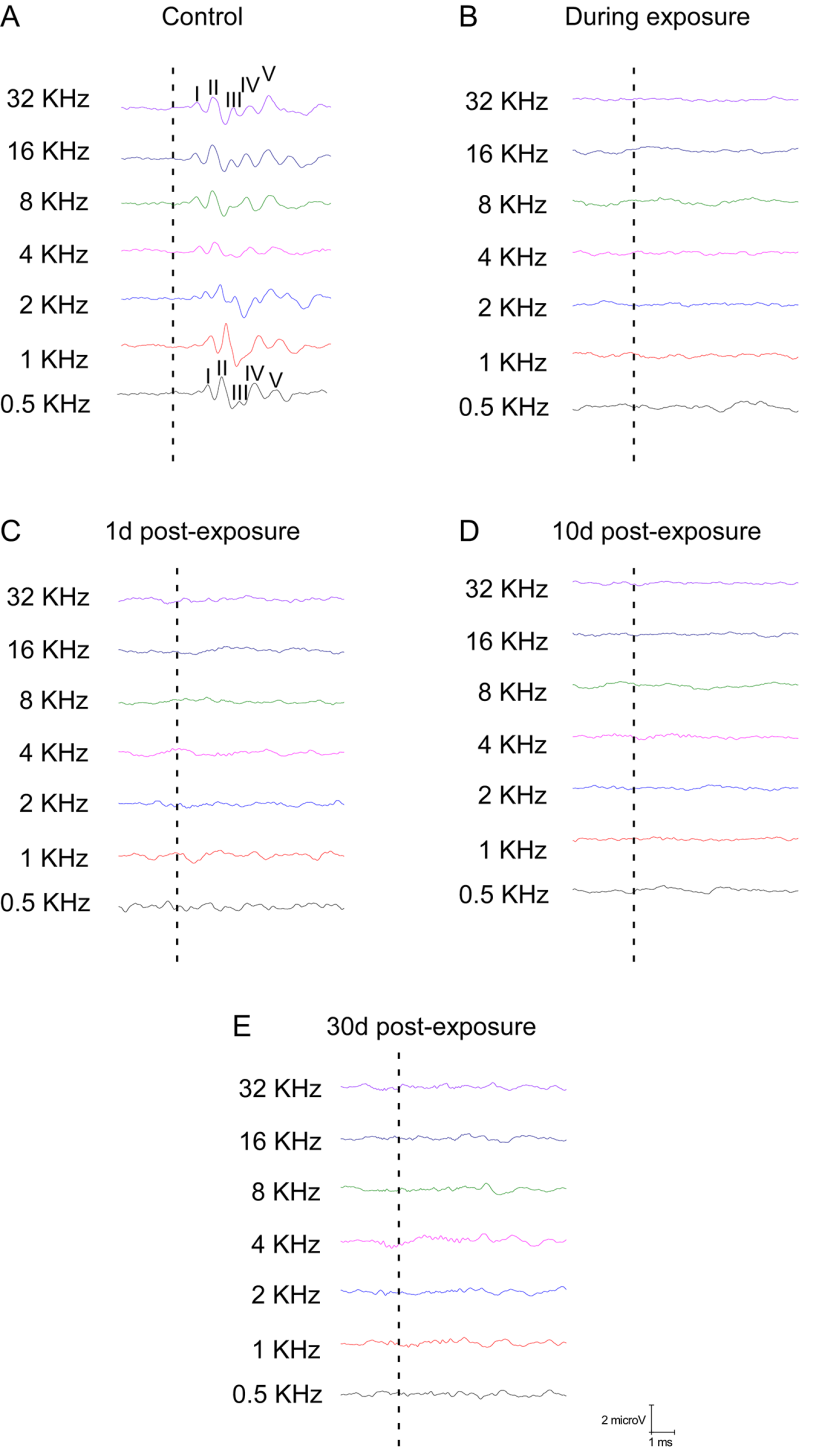
ABR recordings in control and experimental rats. Line graphs depicting examples of ABR recordings in control and experimental animal at 80 dB SPL for all frequencies tested. In the control rats (A) the recordings show a distinctive pattern characterized by 4 to 5 evoked waves after the stimulus onset. On the contrary, in the experimental animals, ABR recordings during exposure (Dur−Exp) (B), 1 day post–exposure (1d−post) (C), 10 days post–exposure (10d−post) (D) and 30 days post–exposure (30d−post) (E) showed a complete loss of evoked waves after stimulus onset at all of the frequencies evaluated, indicating a threshold shift at least up to 30 days after the exposure. The dashed lines indicate stimulus onset.

**Fig 3:**
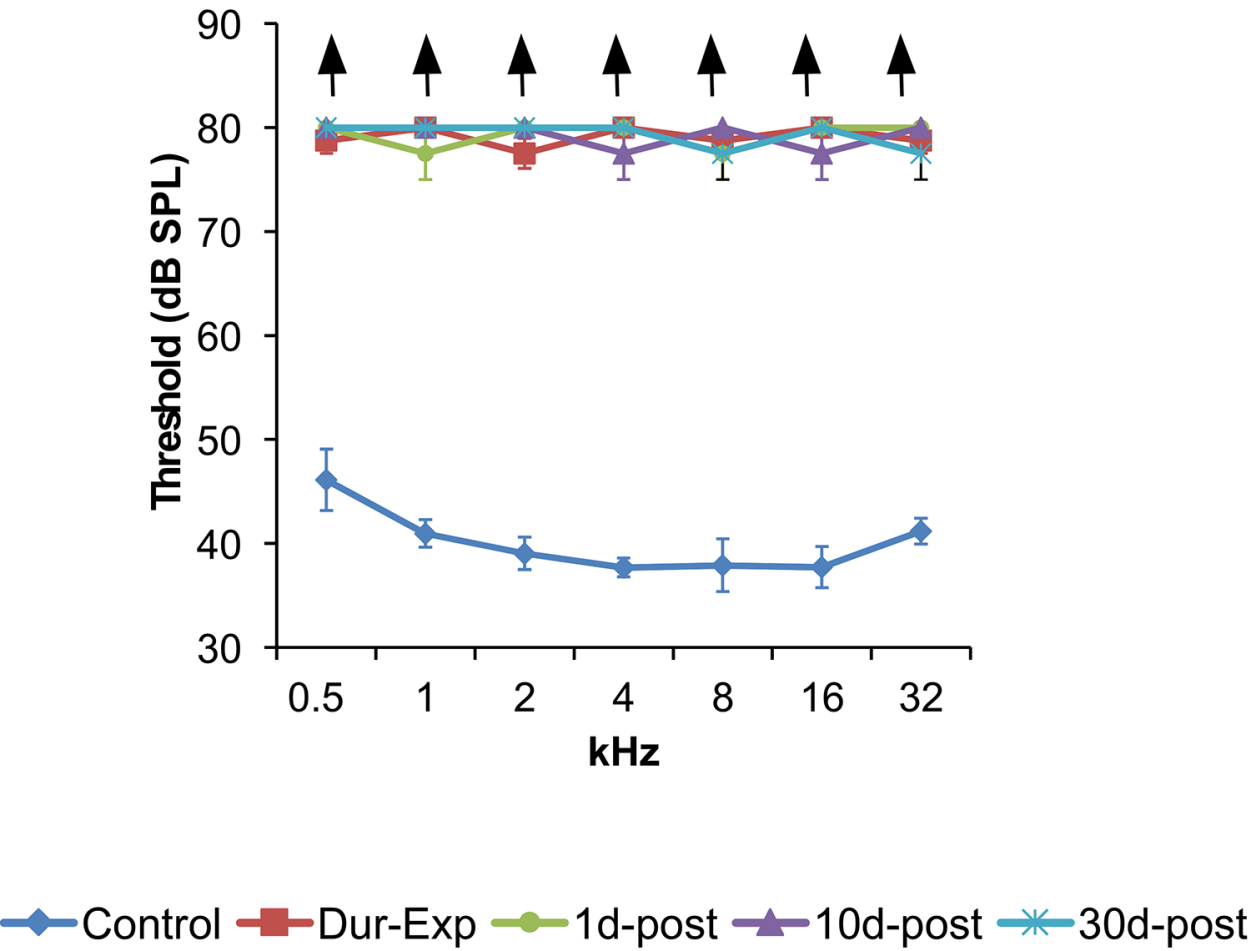
Auditory thresholds in control an experimental animals. Line graphs illustrating auditory thresholds at the frequencies tested in control and experimental animals. In control rats mean thresholds decreased from lower to medium frequencies, to rise again at the highest frequency. Mean values in all experimental groups were high and very similar across frequencies, with average thresholds above 75 dB. Upward arrows indicate that no responses were measured in the noise exposed animals at any frequency.

The ABR recordings of control and experimental animals before noise over stimulation, showed the characteristic pattern of four to five waves after the stimulus onset (Fig 2A). Consistent with previous studies [60,70–74], the largest of all waves was wave II, followed by waves I, IV and V, whereas wave III was the smallest (Fig 2A). On the contrary, recordings in the experimental animals, after the noise exposure, showed complete elimination of the waves at all frequencies evaluated (Fig 2B–2E). The values of the auditory thresholds in the control rats were similar to those described previously for Wistar rats [60,70,72,75,76], with the average thresholds higher at the lowest frequency, decreasing at medium frequencies and increasing again at the highest frequency (Fig 3). In contrast, the auditory thresholds in all experimental groups were high and very similar across frequencies, with mean thresholds above 75 dB (Fig 3).

### Cochlear histopathology

**Fig 4:**
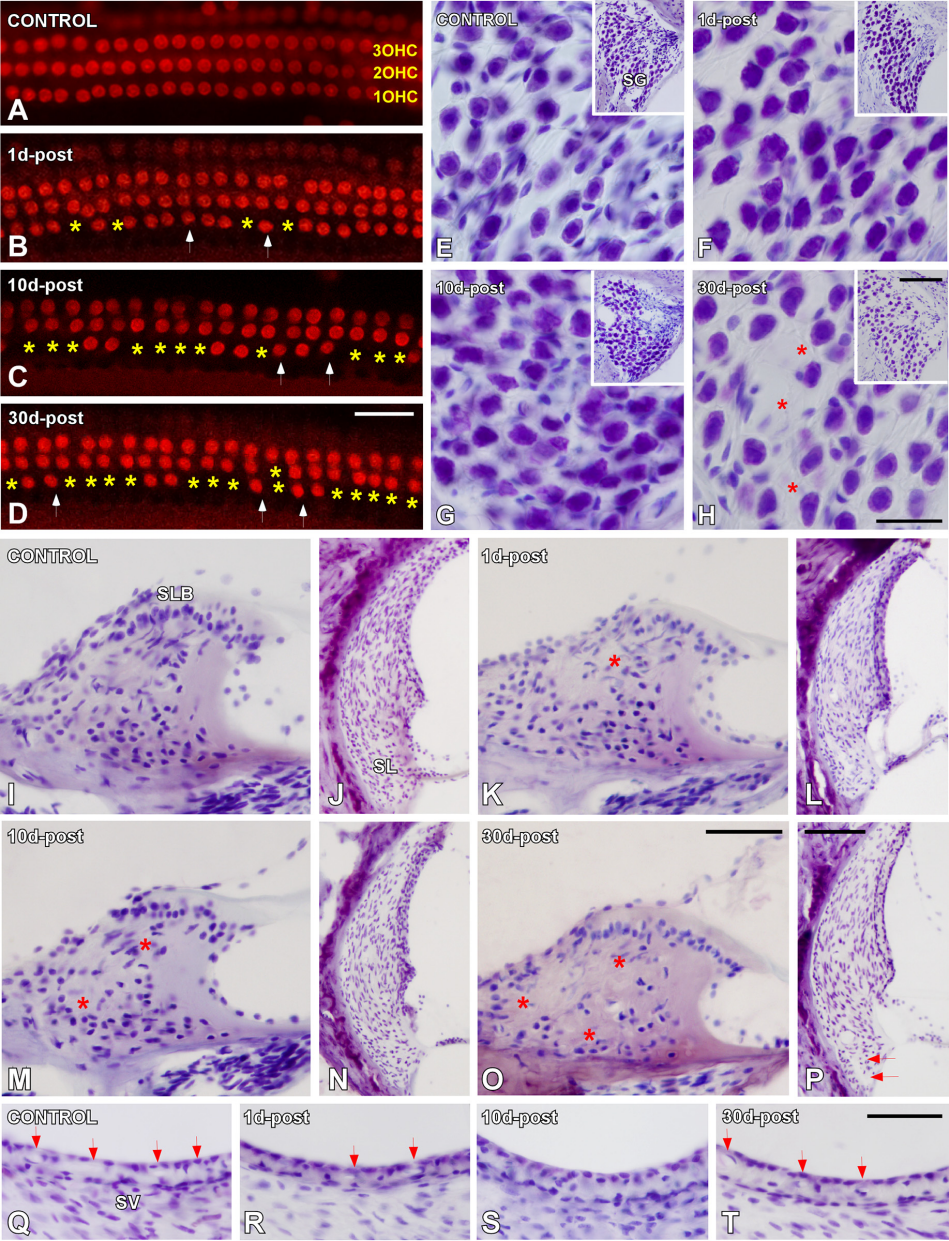
Digital images showing cochlear abnormalities following NIHL. (A–D) Surface preparation images illustrating DAPI nuclear staining in the middle turn region of the organ of Corti in control (A) and noise–exposed (B–D) rats. Yellow asterisks indicate missing OHCs nuclei while arrows point to irregular nuclear contours. Note the greater extent of hair cells damage at 30d post–exposure. (E–H) Nissl–stained cochlear sections showing SGN in unexposed (E) and exposed (F–H) groups. Notice the areas of degeneration particularly at 30d post–exposure (asterisks). (I–P) Nissl–stained cochlear sections showing loss of fibrocytes in the SLB (I, K, M and O) and SL (J, L, N and P) at all time points after the exposure. Asterisks and arrows indicate missing cells. (Q–T) Nissl–stained cochlear sections showing a reduction of the microvasculature of the stria vascularis at 1d (arrows in R) and 10d (S) and partial recovery at 30d (arrows in T) post–exposure in comparison to unexposed rats (arrows in Q). Abbreviations: OHCs, outer hair cells; SG, spiral ganglion; SLB, spiral limbus; SL, spiral ligament; SV, stria vascularis. Scale bars: 25μm in D; 25μm in H; 100μm in H (inset); 50μm in O; 100μm in P; 50μm in T.

Noise–exposed and non–exposed cochleae were analyzed at each time point after trauma to assess the level of cochlear injury and to correlate possible cochlear alterations with the functional deficit observed in the ABR recordings. Surface preparations of the Organ of Corti were used to evaluate the integrity of hair cells. The results showed that outer hair cells (OHCs) damage was associated with survival time in such a way that the degree of missing hair cells nuclei in the cochlea increased with longer survival times after the exposure (yellow asterisks in Fig 4B–4D) when compared to control (Fig 4A) rats. Although there was a regular arrangement of OHCs at all time points after the exposure, some damaged nuclei with irregular shapes were particularly evident (arrows in Fig 4A–4D). The integrity of spiral ganglion neurons (SGN) was also evaluated in mid–modiolar Nissl–stained cochlear sections (Fig 4E–4H). Particularly at day 30 post–exposure, there was a noticeable loss of neurons in addition to areas of degeneration (asterisks in Fig 4H) when compared to the other time points and unexposed rats (Fig 4E–4G). Along with these changes, an overall loss of fibrocytes in both the spiral limbus (asterisks in Fig 4K, 4M and 4O) and the spiral ligament (Fig 4L, 4N and 4P) was also observed at all time points after the exposure in comparison to unexposed (Fig 4I and 4J) rats. Type IV fibrocytes in the cochlear lateral wall were mostly affected at longer time points after the exposure (arrows in Fig 4P). Also, our results demonstrate diminished vascular diameter in the stria vascularis at 1d (arrows in Fig 4R) with a maximum peak at 10d (Fig 4S) post–exposure and a slight recovery at 30d (arrows in Fig 4T) when compared to unexposed (arrows in Fig 4Q) rats.

### Expression profiles of candidate reference genes

**Fig 5:**
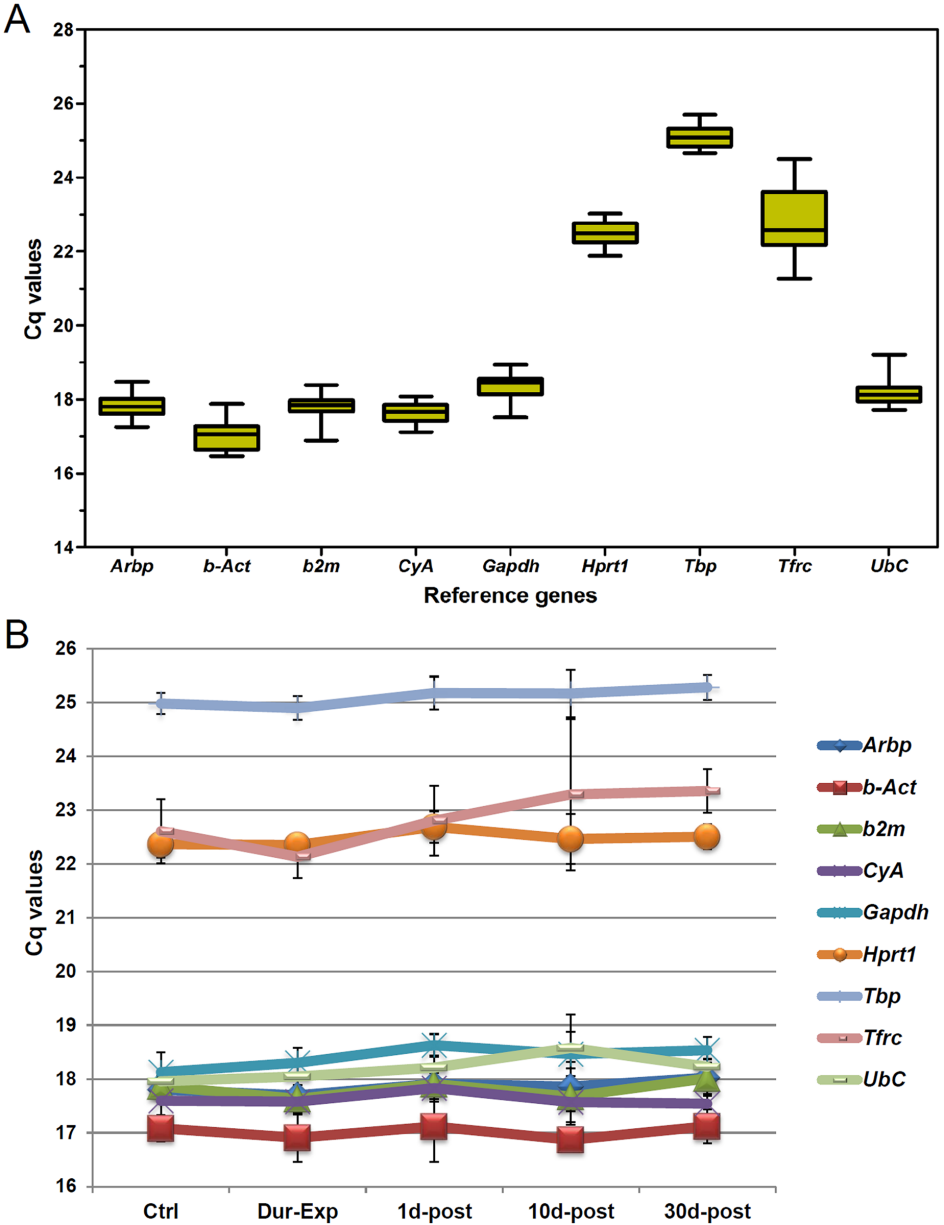
Expression of candidate reference genes. (A) Expression level of nine reference genes in the rat cochlea. The mean Cq values for all the time points of each candidate reference gene is shown as a boxplot representation. The box indicates the 25th and 75th percentiles, the line across the box represents the median and whisker caps show the maximum and minimum values. (B) Expression pattern of each candidate reference gene along the time points. Each data represents the mean ± SD.

The expression of nine candidate reference genes (*Arbp*, *b–Act*, *b2m*, *CyA*, *Gapdh*, *Hprt1*, *Tbp*, *Tfrc* and *UbC*; Table 1) was evaluated in control and experimental cochleae. The raw Cq values were used to calculate the mean Cq for each amplicon in each sample (Fig 5A). The candidate reference genes exhibited Cq values ranging from 17.04 to 25.08. The expression profile of each reference gene along the different time points studied is shown in Fig 5B.

### geNorm analysis

**Fig 6:**
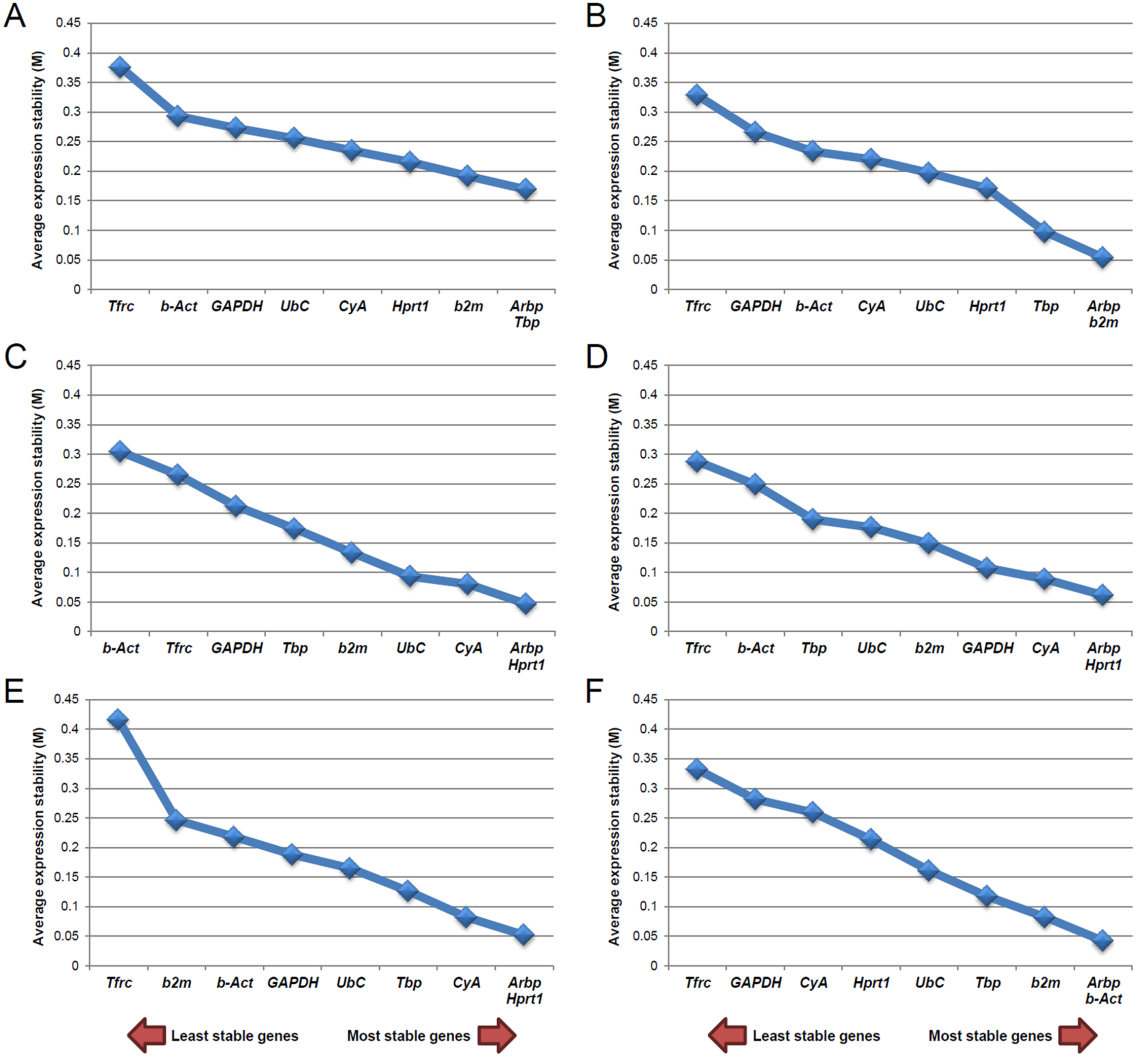
Gene expression stability of the candidate reference genes evaluated by geNorm. Average expression stability values (M) of the nine candidate reference genes plotted from least stable (left) to most stable (right). The geNorm analysis was performed for data from the whole experiment (Total) (A) and from every group independently evaluated: Ctrl (B), Dur–Exp (C), 1d–post (D), 10d–post (E) and 30d–post (F). The most stable genes for the Total experiment were *Tbp/Arbp* and *b2m* and the least stably expressed one was *Tfrc*.

**Fig 7:**
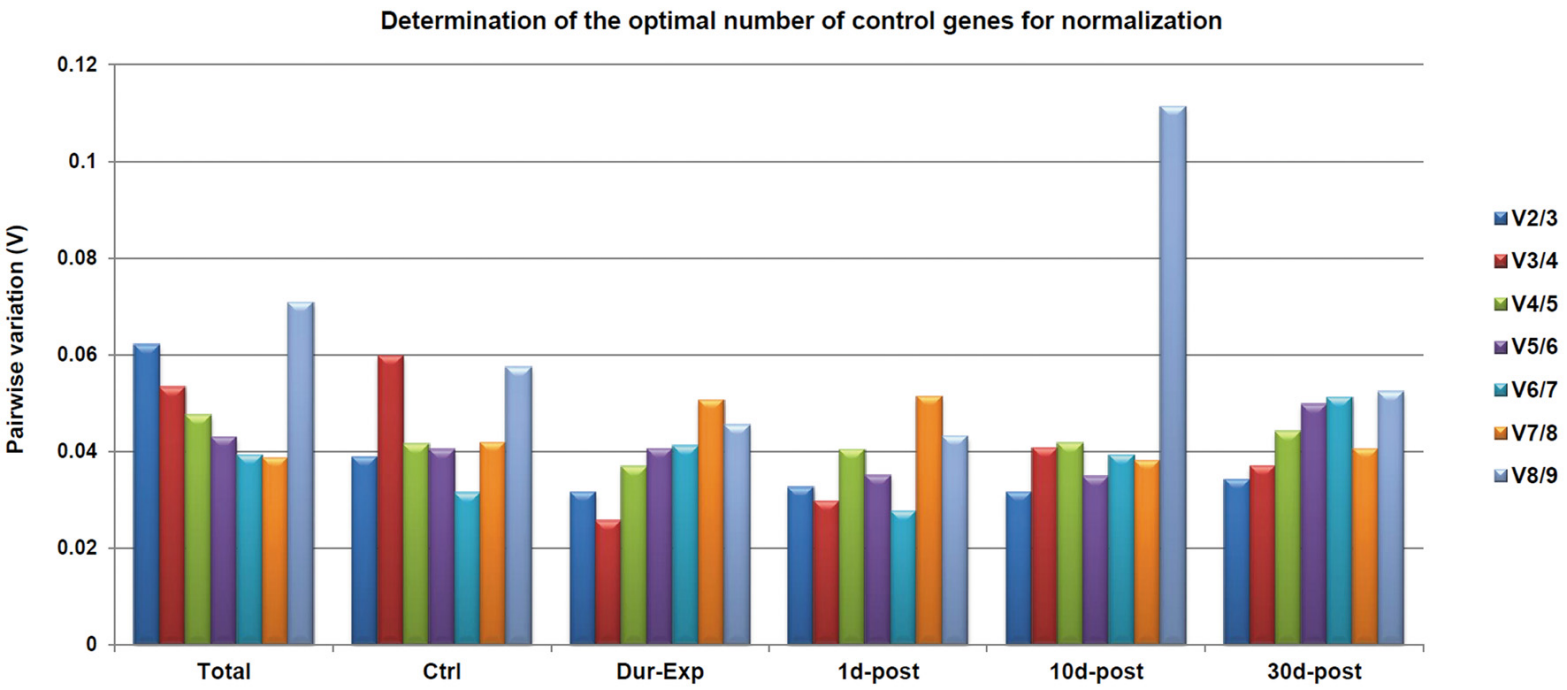
The optimal number of reference genes required for effective normalization as evaluated by geNorm. The pairwise variation (Vn/Vn+1) was caculated by geNorm between the normalization factors NFn and NFn+1 in order to determine the optimal number of reference genes for accurate normalization. The pair variation was evaluated from the Total experiment and from each group independently. In all cases the pairwise variation was < 0.15 cutoff. Thus, the optimal number of reference genes required for normalization is two.

The geNorm algorithm as used to analyze the stability of the nine candidate reference genes in either data from all Ctrl and experimental groups compared together (Total; Fig 6A) or data from every condition analyzed independently (Ctrl, Dur–Exp, 1d–post, 10d–post and 30d–post; Fig 6B–6F). Analysis of the stability value (M) taking into account the whole experiment (Total; Fig 6A) showed that the most stably expressed genes were *Tbp*/*Arbp*, followed by *b2m* (M values of 0.169 and 0.191, respectively; Fig 6A). On the other hand, *Tfrc* was the least stably expressed gene (M value of 0.375; Fig 6A). The results for each experimental condition evaluated independently are also shown in Fig 6B–6F. The pairwise variation (V) was calculated as shown in Fig 7, with V values that were under the 0.15 cut–off for Total and all the experimental conditions independently evaluated. This result indicated that indeed all candidate reference genes were suitable for normalization and could be safely used as endogenous control, although it is recommended to select those most stably expressed [77]. Accordingly, V values revealed that the combination of two reference genes is sufficient for having a suitable normalization. Thus, geNorm analysis indicates that *Tbp*/*Arbp* and *b2m* are the most suitable genes for normalization in our RT–qPCR assays.

### NormFinder analysis

**Fig 8:**
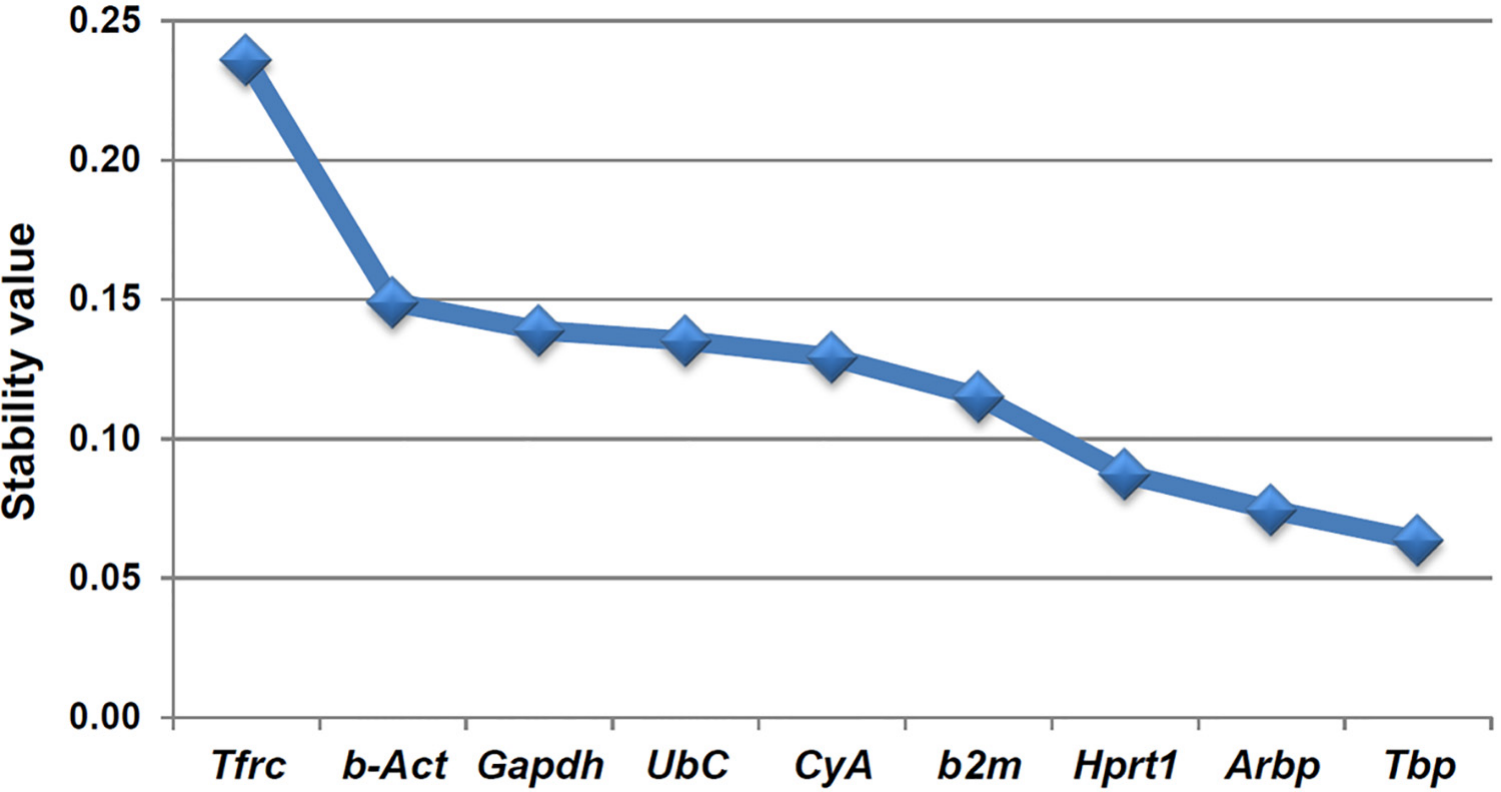
Stability values calculated by NormFinder. The expression stability value (M) of the nine candidate reference genes were calculated using the NormFinder VBA for the Total experiment. The results are plotted from the least stable gene (*Tfrc*) to the most stably expressed one (*Tbp*, M = 0.064).

**Fig 9:**
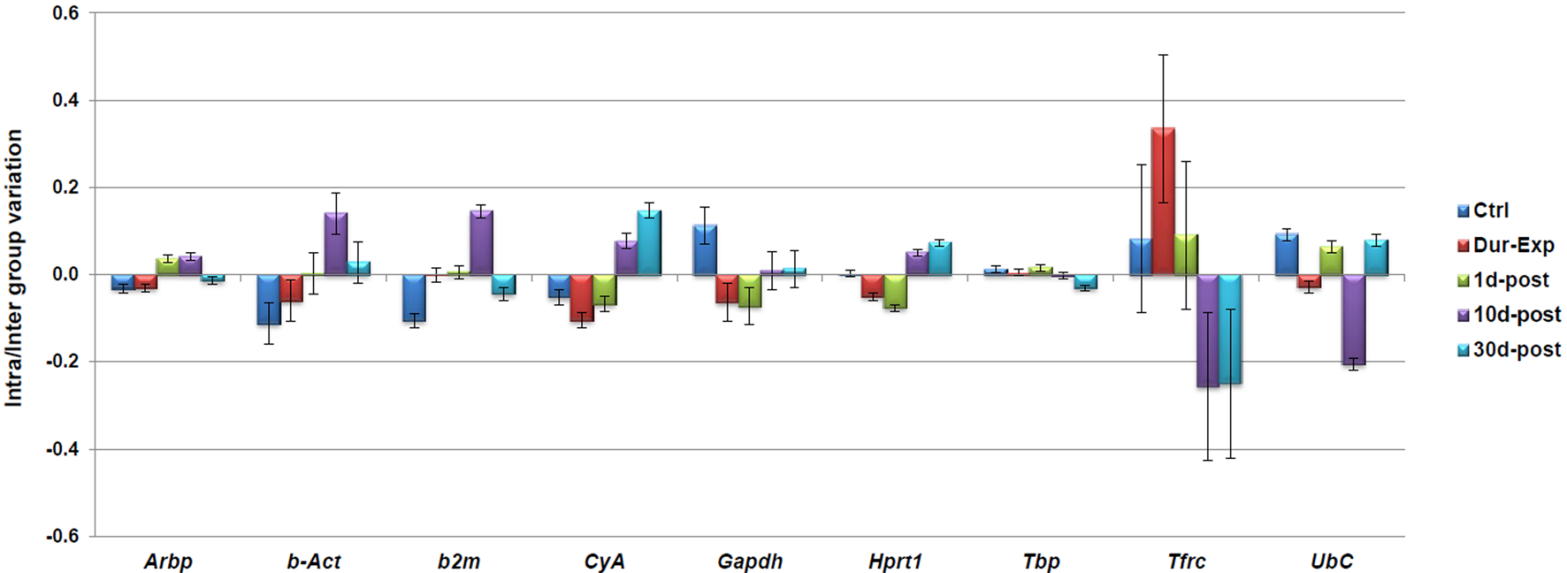
Inter–and Intra–group variation as estimated by NormFinder. The columns represent the inter–group variation and the error bars indicate confidence interval for the inter–group variation for each candidate as the average of the intra–group variances. The top–ranked gene would be that with an inter–group variation as close to zero as possible and at the same time having as small error bars as possible.

In contrast to the geNorm analysis, NormFinder takes into account intra–and inter–group variations and calculates a stability value (M). In the NormFinder analysis, the most stable reference gene (lowest M value) was *Tbp* (M value of 0.064; Fig 8). NormFinder also calculates the stability value for the best combination of two genes. In this case, the best combination was for *Tbp* and *Arbp* (M value of 0.055). On the other hand, the intra–and inter–group variation calculated by NormFinder for every experimental group independently is represented in Fig 9. The graph bars represent the inter–group variances and the error bars the average of the intra–group variances. The top–ranked candidate gene would be that with an inter–group variation as close to zero as possible and at the same time having the smallest error. Thus, NormFinder showed that the best reference gene was *Tbp* while the best combination of two genes was *Tbp* and *Arpb*.

### BestKeeper analysis

In BestKeeper analysis three key data were evaluated: the coefficient of variation (CV), the standard deviation (SD) and the coefficient of correlation (r). As CV and SD give a good estimation of gene stability [59], we decided to rank the reference genes relative to their CV and SD values (Tables 3 and 4). Accordingly, candidate reference gene *Tbp* was the top–ranked and presented also the highest r value (Table 4). Therefore, in agreement with NormFinder and geNorm analyses, BestKeeper revealed that *Tbp* was also the most stable gene whereas *Tfrc* was the least stably expressed.

**Table 3:** Raw output data from the BestKeeper analysis. CP: crossing point = Cq; geo: geometric; ar: arithmetic; min: minimum; max: maximum; std dev: standard deviation; CV: coefficient of variation; coeff. of corr. [r]: coefficient of correlation;

	*Arbp*	*b–Act*	*b2m*	*CyA*	*Gapdh*	*Hprt1*	*Tbp*	*Tfrc*	*UbC*
n	23	23	23	23	23	23	23	23	23
geo Mean [CP]	17.85	17.02	17.82	17.62	18.37	22.46	25.08	22.79	18.17
ar Mean [CP]	17.85	17.03	17.82	17.62	18.37	22.46	25.08	22.81	18.17
min [CP]	17.26	16.46	16.89	17.12	17.51	21.88	24.66	21.26	17.71
max [CP]	18.48	17.88	18.39	18.08	18.93	23.02	25.69	24.49	19.21
**std dev [± CP]**	**0.23**	**0.28**	**0.21**	**0.22**	**0.28**	**0.23**	**0.23**	**0.67**	**0.25**
**CV [% CP]**	**1.32**	**1.66**	**1.17**	**1.24**	**1.53**	**1.05**	**0.92**	**2.96**	**1.36**
min [x–fold]	−1.46	−1.45	−1.94	−1.39	−1.74	−1.48	−1.33	−2.78	−1.35
max [x–fold]	1.51	1.75	1.50	1.35	1.44	1.46	1.52	3.09	2.00
std dev [± x–fold]	1.16	1.20	1.14	1.15	1.20	1.16	1.16	1.55	1.17
**coeff. of corr. [r]**	**0.861**	**0.611**	**0.751**	**0.665**	**0.591**	**0.868**	**0.885**	**0.832**	**0.746**
p–value	0.001	0.002	0.001	0.001	0.003	0.001	0.001	0.001	0.001

**Table 4:** Ranked results from the BestKeeper analysis. CP: crossing point = Cq; CV: coefficient of variation; std dev: standard deviation; coeff. of corr. [r]: coefficient of correlation;

Rank	Gene	CV [% CP]	std dev [± CP]	coeff. of corr. [r]	p–value
**1**	*Tbp*	0.92	0.23	0.885	0.001
**2**	*Hprt1*	1.05	0.23	0.868	0.001
**3**	*b2m*	1.17	0.21	0.751	0.001
**4**	*CyA*	1.24	0.22	0.665	0.001
**5**	*Arbp*	1.32	0.23	0.861	0.001
**6**	*UbC*	1.36	0.25	0.746	0.001
**7**	*Gapdh*	1.53	0.28	0.591	0.003
**8**	*b–Act*	1.66	0.28	0.611	0.002
**9**	*Tfrc*	2.96	0.67	0.832	0.001

The results obtained from geNorm, NormFinder and BestKeeper are summarized in Table 5. All three algorithms suggest that the most stable gene is *Tbp* while the least stably expressed gene is *Tfrc*.

**Table 5:** Summary of the results.

Rank	geNorm	NormFinder	BestKeeper
**1**	*Arbp / Tbp*	*Tbp*	*Tbp*
**2**	*b2m*	*Arbp*	*Hprt1*
**3**	*Hprt1*	*Hprt1*	*b2m*
**4**	*CyA*	*b2m*	*CyA*
**5**	*UbC*	*CyA*	*Arbp*
**6**	*Gapdh*	*UbC*	*UbC*
**7**	*b–Act*	*Gapdh*	*Gapdh*
**8**	*Tfrc*	*b–Act*	*b–Act*
**9**		*Tfrc*	*Tfrc*

### Reference gene validation

**Fig 10:**
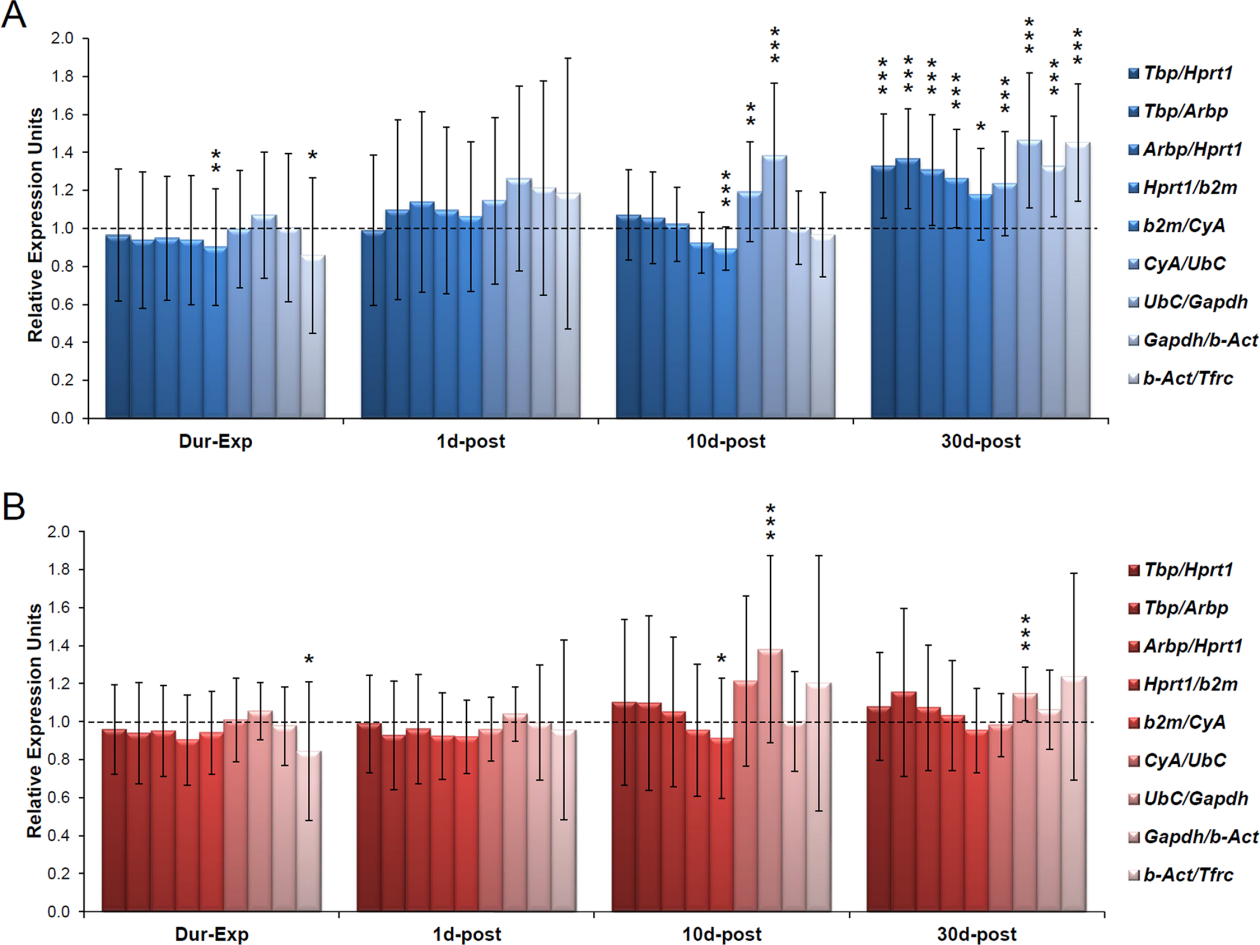
Normalization of *Bad* and *Sod2* gene expression by candidate reference genes in noise–exposed cocheae. (A) *Bad* expression were normalized to nine combinations of reference gene pairs *Tbp*/*Hprt1*, *Tbp/Arbp*, *Arbp/Hprt1*, *Hprt1/b2m*, *b2m/CyA*, *CyA/UbC*, *UbC/Gapdh*, *Gapdh/b–Act* and *b–Act/Tfrc*. A slight up–regulation of *Bad* was observed at 30 days post exposure (30d–post; S2 and S4 Tables). (B) *Sod2* expression referred to nine combinations of reference gene pairs. The results showed similar expression patterns to that in control animals at each time point (S3 and S5 Tables). Data represents the mean ± SD. *p<0.05, **p<0.01, ***p<0.001.

In order to validate the results from the three algorithms and the pairwise variation (V) calculated by geNorm suggesting all reference genes as suitable for normalization, we decided to evaluate the expression of two genes that code for an antioxidant enzyme (*Sod2*) and a pro–apoptotic protein (*Bad*). Both mitochondrial proteins might change their expression as a consequence of noise exposure to regulate oxidative stress and apoptosis [3]. In this regard, we examined the relative expression level of *Bad* and *Sod2* using as endogenous control the geometric mean (as recommended in [57]) of nine combinations of candidate reference gene pairs from the top–ranked to the least stably expressed (see Materials and Methods). As shown in Fig 10A, we observed a significant slight up–regulation (S2 and S4 Tables) of *Bad* at 30d–post for all tested reference gene pairs. At the remaining time points studied (Dur–Exp, 1d–post and10d–post) not important changes were detected relative to control animals (S2 and S4 Tables). On the other hand, the expression pattern of *Sod2* was similar to that in control animals at each time point (Fig 10B; S3 and S5 Tables).

On the other hand, as shown in Fig 10A and 10B, those gene pair combinations composed by the four top–ranked reference genes (*Tbp*/*Hprt1*, *Tbp/Arbp*, *Arbp/Hprt1* and *Hprt1/b2m*; Table 5) showed almost identical results for both target genes at all tested time points (S2 and S3 Tables). Nevertheless, from the fifth reference gene pair (*b2m/CyA*) to the last one (*b–Act/Tfrc*) subtle significant changes (S4 and S5 Tables) were observed at some time points, with the exception of *Gadph/b–Act* that showed similar expression pattern relative to the first four gene combinations. This result suggested that those gene pairs formed by a combination of top–ranked reference genes should be those to be preferentially used for normalizing RT–qPCR assays in the cochlea of Wistar rat and in the context of NIHL.

Finally, when *Bad* and *Sod2* expression were calculated relative to *Tbp* (the most stable gene) taken as single endogenous control, not significant changes were observed (S2–S5 Tables) compared with the reference gene pairs formed by the four top–ranked genes (*Tbp/Hprt1*, *Tbp/Arbp*, *Arbp/Hprt1* and *Hprt1/b2m*). Nevertheless, when the least stably expressed reference gene (*Tfrc*) was taken as single endogenous control, very different results were obtained (S2–S5 Tables). Taken together, these results indicated that *Tbp* could be used as single reference gene for normalizing RT–qPCR assays, whereas *Tfrc* should be preferentially discarded, as suggested by geNorm, NormFinder and BestKeeper.

## Discussion

Recently, numerous studies have employed RT–qPCR for analyzing differential gene expression pattern in the auditory organ after intense noise exposure (for rats see [25–35,37,38]). These studies are essential as they contribute to elucidate the molecular mechanisms that underlie noise–induced cell damage and death in the cochlea and subsequent hearing loss. RT–qPCR remains one of the most sensitive techniques to quantify mRNA [40] but to be so, it is necessary to have a normalization step to minimize the inherent variability [41]. One of the most common ways to normalize RT–qPCR results is the use of reference genes [41,42]. It is well known that among the most commonly used rat strains in biomedical research such as Wistar, Fischer 344, Long Evans or Sprague–Dawley, there are significant genetic and phenotypic differences. Protein expression differences have been recently identified in the cochlea, among Wistar, Sprague–Dawley and Fischer 344 rats with normal hearing function [78]. This confirms that a validation process of candidate reference genes should be performed for every one of these rat strains and in every new experimental design used. In the above studies different reference genes were used for normalization, including *28S* [26], *18S* [28], *CyA* [34], *b–Act* [29,30,37,38], *GAPDH* and *polyubiquitin* [27] and the arithmetic means of *Rpl13a*, *Hprt1* and *b–Act* [31,32] or *Rplp1*, *Hprt1* and *b–Act* [25,33,35]. However, none of them reported a previous validation process of the reference genes used for normalization in the respective rat strain used. In this regard, to our knowledge, there isn’t any systematic study of validation of candidate reference genes for NIHL studies in rat. So, we present here the first study of validation of selected reference genes in the cochleae of control and noise–exposed rats, specifically in Wistar rats and in the context of NIHL.

In the Wistar rat NIHL model reported here, the histological examination of cochlear structures exhibited that almost all these tissues were affected after noise exposure showing a worsen as time progressed. Specifically, we demonstrate that exposure to a continuous broadband white noise at 118 dB SPL for 4 hours, during 4 consecutive days causes a permanent threshold shift which correlates with loss of outer hair cells and spiral ganglion neurons, fibrocytes damage and reduction of cochlear blood supply. Although inner hair cells were not assessed in the present study, we would not expect them to be affected by noise as previous studies have not reported changes in their morphology after noise overstimulation. These corroborates previously published results in different rodent species [2,3,14,79–83] and specifically in Wistar rat by following a different over–exposure protocol [48,84]. On the other hand, although many outer hair cells remained at 30 days following noise overstimulation, deafness is a degenerative condition that may occur without a significant loss of hair cells. Even a transient reduction of cochlear blood supply could vitally damage cochlear tissues and affect cochlear integrity [85].

The nine reference genes used (*Arbp*, *b–Act*, *b2m*, *CyA*, *Gapdh*, *Hprt1*, *Tbp*, *Tfrc* and *UbC*) belonging to different functional groups of genes were selected from the recent literature. The results of the geNorm analysis showed that the most stable reference genes in the cochlea of control and noise–exposed rats were *Tbp*/*Arbp*, followed by *b2m*. The pairwise variation (V) estimated by geNorm revealed that two reference genes were sufficient for accurate normalization. On the other hand, NormFinder showed that the most stably expressed gene was *Tbp* and the best combination of two genes for normalization was *Tbp* and *Arbp*. In agreement to geNorm and NormFinder, BestKeeper detected *Tbp* as the most stably expressed reference gene. Thus, three independent algorithms that employ three different statistical methods resulted in the same output data, that is, *Tbp* as top–ranked reference gene and *Tfrc* as the least stably expressed.

To validate the results, the expression patterns of two mitochondrial genes (*Bad* and *Sod2*) were evaluated. It is postulated that the generation of ROS following noise exposure is involved in cell death in the cochlea and in the pathogenesis of NIHL [3]. In fact, different ROS are detected in the cochlea after noise exposure [4–7]. In this regard, the expression pattern of the pro–apoptotic protein Bad and the antioxidant enzyme Sod2 might be altered after exposure to intense noise. Both genes were analyzed by RT–qPCR and their expression was normalized relative to the geometric mean of different reference gene pair combinations from the top–ranked reference genes to the least stably expressed. No significant changes were observed relative to the Ctrl animals, with the exception of the late up–regulation of *Bad*. Some subtle differences at different time points were observed when the five least stably expressed reference genes were combined, with the exception of *Gapdh/b–Act*. This fact is in agreement with the result of the pairwise variation (V) calculated by geNorm. As stated above, the value of Vn/n+1 for all genes was under a 0.15 cut–off which indicates that indeed all tested reference genes could be used for normalization, although it is recommended to choose the two most stably expressed [77]. Nevertheless, although any reference gene pairs could be theoretically selected and potentially give quantitatively similar results when analyzing *Bad* and *Sod2* expression, subtle changes in the results would be expected as we move towards the least stably expressed reference genes. In this way, those combinations formed by the most stable reference genes (*Tbp*, *Arbp*, *Hprt1* and *b2m*) are the most strongly recommended to be used for homogenous and accurate results whereas the others should be discarded, especially the least stably expressed gene (*Tfrc*). *Tbp* is also the only one stated as the best reference gene by the three independent applications (geNorm, NormFinder and BestKeeper). In fact, when *Tbp* was used as single endogenous control for normalizing *Bad* and *Sod2* expression, similar results were observed compared with those combinations composed by the top–ranked reference genes (*Tbp*, *Arbp*, *Hprt1* and *b2m*). This suggests that *Tbp* could be taken as single normalizing gene for RT–PCR assays in the cochlea of Wistar rat and in the context of NIHL. Similarly, a recent study has also shown *Tbp* as a good reference gene in other post traumatic tissues [86].

On the other hand, these results are, in agreement with recent literature showing expression patterns of Bad protein in the cochlea of noise–exposed mice [36]. After noise exposure, an early increase in the expression of the activated form of Bad (P–Bad) was detected in OHCs, but not of the total–Bad protein, which was redistributed [36]. Therefore, other key apoptosis regulators could be good candidates for RT–qPCR analysis in response to NIHL, such as *Bcl–xl* and *Bak* genes. Indeed, Bcl–xl and Bak proteins are up–regulated at 1 day after noise exposure in guinea pigs after different noise exposure protocols [39]. The mRNA of *Bcl–2*, *Bax* and *Bcl–x* is also detected in the developing mouse cochlea at different ages [87].

On the second place, our results are in contrast with recent literature showing an increase in the expression of Sod2 protein after noise exposure in mice [36]. However, it is important to note that not always there is a concordance between the mRNA and protein expression data [88–90] and that other antioxidant enzymes in the cell could also be relevant as scavenger molecules during the pathological process of NIHL such as Sod1, Catalase and Glutathione peroxidases (GPx1, GPx2 or GPx3). In this regard, not only Sod2 protein is expressed in the cochlea [36,91–93], but also Sod1 [92] and GPx1 [94] proteins exhibit extensive expression along cochlear structures.

Finally, in the context of ARHL, a previous report showed that *Gapdh* was the most stably expressed between the cochleae of young and old Fischer 344 rats [45]. Here we showed that *Gadph* is one of the least stably expressed genes in Wistar rats during NIHL. We have used only young animals (3–months old) and the age is not a variable to take into account among the individuals from our experiments. According to pairwise variation data calculated by geNorm, *Gapdh* could also be used for normalization. However, only *Gapdh/b–Act* gene pair combination displayed similar *Bad* and *Sod2* expression patterns relative to the combinations of *Tbp*, *Arbp*, *Hprt1* and *b2m* genes, whereas *UbC/Gapdh* showed variations relative to the combinations of these top–ranked genes. Thus *Gapdh* should be preferentially discarded in an experimental model of NIHL that use Wistar rat as animal model.

The present study should be helpful to elucidate molecular mechanisms that underlie NIHL, when using RT–qPCR gene expression assays. Indeed, it provides the first essential step for all further RT–qPCR studies that use Wistar rat as an animal model in the context of NIHL. In conclusion we have demonstrated that *Tbp* is the most stable reference gene in the Wistar rat cochlea during NIHL experiments and *Tfrc* is the least stably expressed.

## Supporting Information

S1 Fig Specificity of gene primer pairs. RT–qPCR melting curves are shown with one primer pair per panel. Reference gene primer names are indicated in the title of each panel.
(TIF)

S1 Table MIQE checklist. (PDF)

S2 Table Relative expression level (mean and standard deviation) of *Bad* relative to different reference genes or reference gene pairs in Control and Experimental samples. (PDF)

S3 Table Relative expression level (mean and standard deviation) of *Sod2* relative to different reference genes or reference gene pairs in Control and Experimental samples. (PDF)

S4 Table Statistical analysis of *Bad* gene expression changes relative to different reference genes or reference gene pairs. (PDF)

S5 Table Statistical analysis of *Sod2* gene expression changes relative to different reference genes or reference gene pairs. (PDF)
