# Supplementary figures and images for: Validation of Reference Genes for RT–qPCR Analysis in Noise–Induced Hearing Loss: A Study in Wistar Rat
Source: PLoS One. 2015 Sep 14;10(9):e0138027. doi: 10.1371/journal.pone.0138027 (PMC4569353; doi:10.1371/journal.pone.0138027)

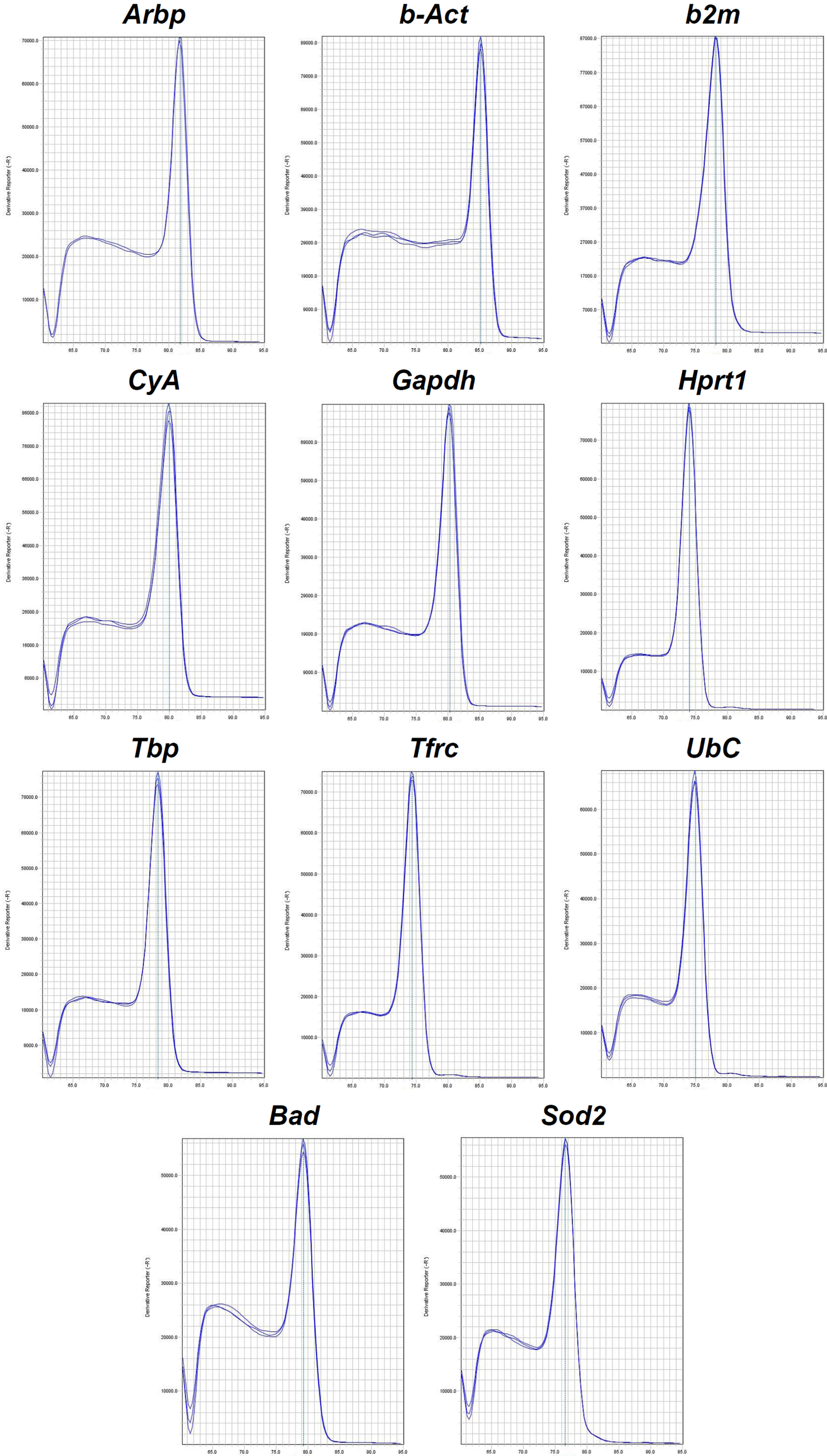

Supplement: S1 Fig — RT–qPCR melting curves are shown with one primer pair per panel. Reference gene primer names are indicated in the title of each panel. (TIF) [file pone.0138027.s001.tif]
