# Supplementary material for: Validation of Reference Genes for RT–qPCR Analysis in Noise–Induced Hearing Loss: A Study in Wistar Rat
Source: PLoS One. 2015 Sep 14;10(9):e0138027. doi: 10.1371/journal.pone.0138027 (PMC4569353; doi:10.1371/journal.pone.0138027)
Supplement: S1 Table — (PDF) [file pone.0138027.s002.pdf]

S1 Table. MIQE checklist.

ITEM TO CHECK	IMPORTANCE	DESCRIPTION
**EXPERIMENTAL DESIGN**		
Definition of experimental and control groups	**E**[a]	Animals with positive Preyer's reflex and normal ABRs were randomly assigned to one of the following groups: Control (Ctrl), during exposure (Dur−Exp), 1 day post–exposure (1d−post), 10 days post–exposure (10d−post) and 30 days post–exposure (30d−post).
Number within each group	**E**	Control (Ctrl; n=7), during exposure (Dur−Exp; n=4), 1 day post–exposure (1d−post; n=4), 10 days post–exposure (10d−post; n=4) and 30 days post–exposure (30d−post; n=4).
Assay carried out by core lab or investigator's lab?	D[a]	Assay carried out by investigator's lab.
Acknowledgement of authors' contributions	D	Described in the manuscript.
**SAMPLE**		
Description	**E**	**Experimental samples:** whole cochleae of noise–exposed animals sacrificed at different time points. **Control samples:** whole cochleae of non–exposed animals sacrificed at different time points.
Volume/mass of sample processed	D	
Microdissection or macrodissection	**E**	Microdissection.
Processing procedure	**E**	Animals were deeply anesthetized with 1.5–2% isoflurane (1 L/min O_2 flow rate) followed by an intraperitoneal injection of ketamine (80mg/kg) and xylazine (10mg/kg). After euthanasia, temporal bones were rapidly removed and placed in cold 1X Phosphate Buffer Saline (PBS). Whole cochleae were isolated using a dissection microscope, collected into cryotubes.
If frozen – how and how quickly?	**E**	Samples were immediately frozen on dry ice. The whole process was carried out within 6–8min.
If fixed – with what, how quickly?	**E**	Not fixed.
Sample storage conditions and duration (especially for FFPE samples)	**E**	Samples were stored at –80°C.
**NUCLEIC ACID EXTRACTION**		
Procedure and/or instrumentation	**E**	Frozen cochleae were weighed and the corresponding volume of cold TRIzol reagent (Life Technologies) was added according to manufacturer's instructions. Cochleae were then quickly homogenized using a Polytron PT 2100 homogenizer (Kinematica, Dispersing aggregate PT−DA 2105/2EC; Rotor–Ø 3mm) at 30x1000 rpm for < 30 seconds. Total RNA was extracted according to TRIzol manufacturer's instructions. The purified RNA was dissolved in 30 µl RNAse free water and stored at –80°C.
Name of kit and details of any modifications	**E**	TRIzol Reagent (Life Technologies). We exactly followed manufacture's protocol.
Source of additional reagents used	D	
Details of DNase or RNAse treatment	**E**	No DNase treatment was performed.

Contamination assessment (DNA or RNA)	**E**	Non reverse transcriptase (–RT) controls were performed in order to determine genomic DNA contamination of the RNA samples by using the *UbC* gene primers. These were the only ones that amplified the same region in both the cDNA and the genomic DNA (see Table 2). The average difference among the *UbC* expression in each sample and the corresponding –RT expression was 14.25 ± 1.09 Cq indicating that no genomic DNA was present in the RNA samples.
Nucleic acid quantification	**E**	Quantity of RNAs were assessed by electrophotometric assay.
Instrument and method	**E**	Nanodrop ND−1000 (Thermo Scientific).
Purity (A260/A280)	D	RNA purity was determined by measuring the absorbance ratio 260/280.
Yield	D	
RNA integrity method/instrument	**E**	Electrophoretic analysis (0.8% agarose gels; 60V) was performed in order to examine RNA integrity. All RNA samples showed suitable A260/A280 and 28S/18S ratios.
RIN/RQI or Cq of 3' and 5' transcripts	**E**	Not applicable.
Electrophoresis traces	D	
Inhibition testing (Cq dilutions, spike or other)	**E**	The standard curve was considered sufficient to rule out the presence of inhibitors of reverse–transcription activity or qPCR.
**REVERSE TRANSCRIPTION**		
Complete reaction conditions	**E**	Reaction conditions were as follows: 65°C for 5 min, 37°C for 5 min, 42°C for 1h, 72°C for 10 min, 4°C for ∞.
Amount of RNA and reaction volume	**E**	Amount of RNA: 1 µg. Reaction volume: 20 µl.
Priming oligonucleotide (if using GSP) and concentration	**E**	Oligo(dT)$_{18}$ was used as primer at a final concentration of 5 µM.
Reverse transcriptase and concentration	**E**	RevertAid RT (Thermo Scientific). Final concentration: 10 U/µl.
Temperature and time	**E**	Specified in "Complete reaction conditions".
Manufacturer of reagents and catalogue numbers	D	RevertAid First Strand cDNA Synthesis Kit (Thermo Scientific, Cat. No. #K1622).
Cqs with and without RT	D	
Storage conditions of cDNA	D	cDNAs were stored at 4°C for immediate use or at –20°C for long–term storage.
**qPCR TARGET INFORMATION**		
If multiplex, efficiency and LOD of each assay.	**E**	Not applicable.
Sequence accession number	**E**	Specified at Table 2.
Location of amplicon	D	
Amplicon length	**E**	Specified at Table 2.
In silico specificity screen (BLAST, etc)	**E**	Gene specificities were tested by BLAST analysis (NCBI) showing 100% homology for all tested genes.
Pseudogenes, retropseudogenes or other homologs?	D	None found.

Sequence alignment	D	
Secondary structure analysis of amplicon	D	Secondary structure of amplicon was analized by using OligoAnalizer v1.2 and OligoExplorer v1.2 softwares.
Location of each primer by exon or intron (if applicable)	**E**	Specified at Table 2.
What splice variants are targeted?	**E**	No splice variants have been described for each tested genes.
**qPCR OLIGONUCLEOTIDES**		
Primer sequences	**E**	Specified at Table 2.
RTPrimerDB Identification Number	D	Not applicable.
Probe sequences	D	Not applicable.
Location and identity of any modifications	**E**	Location specified at Table 2. Any oligonucleotide was modified.
Manufacturer of oligonucleotides	D	Thermo Scientific.
Purification method	D	HPSF.
**qPCR PROTOCOL**		
Complete reaction conditions	**E**	RT–qPCRs were performed in a One Step Plus Real–Time PCR System machine (Applied Biosystems) using 96–well plates and Fast SYBR Green Master Mix (Applied Biosystems, Cat. No. 4385612) as reagent. Briefly, the RT–qPCR reaction mix per well consisted of 2.8µl of sterile H2O MilliQ, 0.1µl of each primer (final concentration of 100nM), 5µl of Fast SYBR Green Master Mix and 2µl of 1:10–diluted cDNA. After the reaction mix was dispensed in the corresponding wells, the plate was centrifuged at 1200 rpm for 2 min. The RT–qPCR amplification was performed starting with an initial activation step (95°C for 20 s) followed by 40 cycles of 95°C for 6 s and 60°C for 45 s.
Reaction volume and amount of cDNA/DNA	**E**	Reaction volume: 10 µl. Amount of cDNA: 2µl of 1:10–diluted cDNA.
Primer, (probe), Mg++ and dNTP concentrations	**E**	Primer final concentration: 100 nM. Mg^{2+} and dNTP were included in the Fast SYBR Green Master Mix (Applied Biosystems).
Polymerase identity and concentration	**E**	Included in the Fast SYBR Green Master Mix (Applied Biosystems, Cat. No. 4385612).
Buffer/kit identity and manufacturer	**E**	Fast SYBR Green Master Mix (Applied Biosystems, Cat. No. 4385612).
Exact chemical constitution of the buffer	D	
Additives (SYBR Green I, DMSO, etc.)	**E**	SYBR Green I.
Manufacturer of plates/tubes and catalog number	D	MicroAmp® Fast Optical 96–Well Reaction Plate with Barcode (Applied Biosystems, Cat. No. 4346906) and MicroAmp® Optical Adhesive Film (Applied Biosystems, Cat. No. 4360954).
Complete thermocycling parameters	**E**	The RT–qPCR amplification was performed starting with an initial activation step (95°C for 20 s) followed by 40 cycles of 95°C for 6 s and 60°C for 45 s.
Reaction setup (manual/robotic)	D	Manual.
Manufacturer of qPCR instrument	**E**	One Step Plus Real–Time PCR System machine (Applied Biosystems).

qPCR VALIDATION		
Evidence of optimisation (from gradients)	D	
Specificity (gel, sequence, melt, or digest)	**E**	The melting curve was generated by an initial denaturation step (95°C for 20 s) followed by a gradual heating from 60°C to 95°C (ramp of 0.3°C).
For SYBR Green I, Cq of the NTC	**E**	Non–template controls (NTC) generated Cq values >35.
Standard curves with slope and y–intercept	**E**	
PCR efficiency calculated from slope	**E**	Specified at Table 2.
Confidence interval for PCR efficiency or standard error	D	
r2 of standard curve	**E**	Specified at Table 2.
Linear dynamic range	**E**	In average, linear dynamic range was considered taking into account the linearity of the standard curves; from five 10–fold serial dilutions of a control cochlea cDNA.
Cq variation at lower limit	**E**	
Confidence intervals throughout range	D	
Evidence for limit of detection	**E**	
If multiplex, efficiency and LOD of each assay.	**E**	Not applicable.
**DATA ANALYSIS**		
qPCR analysis program (source, version)	**E**	Step One Software v2.3 (Applied Biosystems).
Cq method determination	**E**	Cq was calculated by determining the Threshold. The same threshold was taken for all genes and assays.
Outlier identification and disposition	**E**	None of the Cq values was discarded.
Results of NTCs	**E**	Non–template controls (NTC) generated Cq values >35.
Justification of number and choice of reference genes	**E**	The aim of the study was to validate reference genes. In this regard, they were researched from the recent literature. Careful attention was paid to select those genes from different functional groups. Theoretically this should reduce the chance that they might be co–regulated
Description of normalisation method	**E**	Described in Materials and Methods.
Number and concordance of biological replicates	D	
Number and stage (RT or qPCR) of technical replicates	**E**	The experiments were performed technically in triplicate and biologically in quadruplicate with the exception of the Ctrl group that contained seven biological samples.
Repeatability (intra–assay variation)	**E**	Mean standard deviation of technical triplicates: 0.045.
Reproducibility (inter–assay variation, %CV)	D	
Power analysis	D	

Statistical methods for result significance	**E**	Depending on whether the sample distributions met the assumptions of normality based on the Kolmogorov–Smirnov test of normality, Student's t test was used to compare different groups of data. When the assumption was not met, the Mann–Whitney test was used.
Software (source, version)	**E**	GraphPad Prism 5.0 (GraphPad Software Inc).
Cq or raw data submission using RDML	D	

[a] (E) essential and (D) desirable information to be included in research reports using qPCR.
