# Supplementary material for: Validation of Reference Genes for RT–qPCR Analysis in Noise–Induced Hearing Loss: A Study in Wistar Rat
Source: PLoS One. 2015 Sep 14;10(9):e0138027. doi: 10.1371/journal.pone.0138027 (PMC4569353; doi:10.1371/journal.pone.0138027)
Supplement: S2 Table — (PDF) [file pone.0138027.s003.pdf]

S2 Table. Relative expression level (mean and standard deviation) of *Bad* relative to different reference genes or reference gene pairs in Control and Experimental samples.

Bad		*Tbp*	*Tbp/Hprt1*	*Tbp/Arbp*	*Arbp/Hprt1*	*Hprt1/b2m*	*b2m/CyA*	*CyA/UbC*	*UbC/Gapdh*	*Gapdh/b−Act*	*b−Act/Tfrc*	*Tfrc*
**MEAN**	**Ctrl**	1.02	1.02	1.02	1.02	1.00	1.01	1.00	1.03	1.01	1.03	1.11
	**Dur−Exp**	0.96	0.96	0.94	0.95	0.94	0.90	1.00	1.07	1.00	0.86	0.78
	**1d−post**	1.14	0.99	1.10	1.14	1.10	1.06	1.15	1.26	1.21	1.18	1.32
	**10d−post**	1.11	1.07	1.06	1.02	0.92	0.89	1.19	1.38	1.00	1.12	1.93
	**30d−post**	1.40	1.33	1.37	1.31	1.26	1.18	1.24	1.46	1.33	1.45	1.88
**ST. DEV.[a]**	**Ctrl**	0.20	0.18	0.22	0.19	0.17	0.10	0.14	0.24	0.14	0.25	0.52
	**Dur−Exp**	0.38	0.35	0.36	0.33	0.34	0.31	0.31	0.33	0.39	0.41	0.39
	**1d−post**	0.50	0.40	0.47	0.47	0.44	0.39	0.44	0.49	0.57	0.71	0.92
	**10d−post**	0.30	0.24	0.24	0.20	0.16	0.11	0.26	0.38	0.19	0.35	1.26
	**30d−post**	0.22	0.27	0.26	0.29	0.26	0.24	0.27	0.35	0.26	0.31	0.63
	MEAN[b] ►	**0.35**	**0.31**	**0.33**	**0.32**	**0.30**	**0.26**	**0.32**	**0.39**	**0.35**	**0.45**	**0.80**

[a]ST. DEV.: Standard deviation; [b]*MEAN* corresponds to the average of ST. DEV. of experimental groups.
