# Supplementary material for: Validation of Reference Genes for RT–qPCR Analysis in Noise–Induced Hearing Loss: A Study in Wistar Rat
Source: PLoS One. 2015 Sep 14;10(9):e0138027. doi: 10.1371/journal.pone.0138027 (PMC4569353; doi:10.1371/journal.pone.0138027)
Supplement: S3 Table — (PDF) [file pone.0138027.s004.pdf]

S3 Table. Relative expression level (mean and standard deviation) of *Sod2* relative to different reference genes or reference gene pairs in Control and Experimental samples.

	Sod2	***Tbp***	***Tbp/Hprt1***	***Tbp/Arbp***	***Arbp/Hprt1***	***Hprt1/b2m***	***b2m/CyA***	***CyA/UbC***	***UbC/Gapdh***	***Gapdh/b−Act***	***b−Act/Tfrc***	***Tfrc***
**MEAN**	**Ctrl**	1.07	1.05	1.08	1.06	1.05	1.05	1.02	1.01	1.02	1.10	1.19
	**Dur−Exp**	0.96	0.96	0.94	0.95	0.90	0.94	1.01	1.06	0.98	0.84	0.77
	**1d−post**	0.96	0.99	0.93	0.96	0.92	0.92	0.96	1.04	0.99	0.95	1.05
	**10d−post**	1.16	1.10	1.10	1.05	0.95	0.91	1.21	1.38	1.00	1.20	2.18
	**30d−post**	1.17	1.08	1.15	1.07	1.03	0.95	0.98	1.15	1.06	1.24	1.71
**ST. DEV.[a]**	**Ctrl**	0.41	0.33	0.44	0.36	0.34	0.29	0.22	0.16	0.22	0.51	0.74
	**Dur−Exp**	0.28	0.23	0.27	0.24	0.24	0.22	0.22	0.15	0.21	0.37	0.36
	**1d−post**	0.27	0.26	0.29	0.29	0.23	0.19	0.17	0.14	0.30	0.47	0.56
	**10d−post**	0.53	0.44	0.46	0.39	0.35	0.31	0.45	0.49	0.26	0.67	1.83
	**30d−post**	0.40	0.28	0.44	0.33	0.29	0.22	0.16	0.14	0.21	0.55	0.87
	MEAN[b] ►	**0.37**	**0.30**	**0.36**	**0.31**	**0.28**	**0.24**	**0.25**	**0.23**	**0.24**	**0.51**	**0.90**

[a]ST.DEV.: Standard deviation; [b]*MEAN* corresponds to the average of ST. DEV. of experimental groups.
