# Supplementary material for: Validation of Reference Genes for RT–qPCR Analysis in Noise–Induced Hearing Loss: A Study in Wistar Rat
Source: PLoS One. 2015 Sep 14;10(9):e0138027. doi: 10.1371/journal.pone.0138027 (PMC4569353; doi:10.1371/journal.pone.0138027)
Supplement: S4 Table — (PDF) [file pone.0138027.s005.pdf]

S4 Table. Statistical analysis of *Bad* gene expression changes relative to different reference genes or reference gene pairs.

Reference genes	Statistical test	Dur–Exp	1d–post	10d–post	30d–post
Tbp	Mann Whitney test	U=632.0 p=NS[a]	U=681.0 p=NS	U=583.0 p=NS	U=137.0 p<0.001
Tbp/Hprt1	Mann Whitney test	U=622.0 p=NS	U=383.0 p=NS	U=600.0 p=NS	U=230.0 p<0.001
Tbp/Arbp	Mann Whitney test	U=567.0 p=NS	U=679.0 p=NS	U=617.0 p=NS	U=225.0 p<0.001
Arbp/Hprt1	Mann Whitney test	U=563.0 p=NS	U=653.0 p=NS	U=670.0 p=NS	U=278.0 p<0.001
Hprt1/b2m	Student's t–test	t=1.271 df=75 p=NS	t=1.367 df=75 p=NS	t=1.909 df=75 p=NS	t=5.332 df=75 p<0.001
b2m/CyA	Mann Whitney test	U=404.0 p<0.01	U=678.0 p=NS	U=302.0 p<0.001	U=468.0 p<0.05
CyA/UbC	Mann Whitney test	U=574.0 p=NS	U=664.0 p=NS	U=387.0 p<0.01	U=335.0 p<0.001
UbC/Gapdh	Mann Whitney test	U=664.0 p=NS	U=548.0 p=NS	U=290.0 p<0.001	U=218.0 p<0.001
Gapdh/b-Act	Mann Whitney test	U=666.0 p=NS	U=674.0 p=NS	U=674.0 p=NS	U=218.0 p<0.001
β–Act/Tfrc	Mann Whitney test	U=496.0 p<0.05	U=678.0 p=NS	U=597.0 p=NS	U=200.0 p<0.001
Tfrc	Mann Whitney test	U=436.0 p<0.01	U=682.0 p=NS	U=430.0 p<0.01	U=218.0 p<0.001

[a]NS: non–significant.
