# Supplementary material for: Validation of Reference Genes for RT–qPCR Analysis in Noise–Induced Hearing Loss: A Study in Wistar Rat
Source: PLoS One. 2015 Sep 14;10(9):e0138027. doi: 10.1371/journal.pone.0138027 (PMC4569353; doi:10.1371/journal.pone.0138027)
Supplement: S5 Table — (PDF) [file pone.0138027.s006.pdf]

S5 Table. Statistical analysis of *Sod2* gene expression changes relative to different reference genes or reference gene pairs.

Reference genes	Statistical test	Dur–Exp	1d–post	10d–post	30d–post
Tbp	Mann Whitney test	U=592.0 p=NS[a]	U=608.0 p=NS	U=654.0 p=NS	U=602.0 p=NS
Tbp/Hprt1	Mann Whitney test	U=594.0 p=NS	U=638.0 p=NS	U=683.0 p=NS	U=651.0 p=NS
Tbp/Arbp	Mann Whitney test	U=591.0 p=NS	U=566.0 p=NS	U=677.0 p=NS	U=618.0 p=NS
Arbp/Hprt1	Mann Whitney test	U=614.0 p=NS	U=585.0 p=NS	U=668.0 p=NS	U=647.0 p=NS
Hprt1/b2m	Mann Whitney test	U=525.0 p=NS	U=540.0 p=NS	U=570.0 p=NS	U=680.0 p=NS
b2m/CyA	Mann Whitney test	U=569.0 p=NS	U=511.0 p=NS	U=488.0 p<0.05	U=529.0 p=NS
CyA/UbC	Mann Whitney test	U=679.0 p=NS	U=583.0 p=NS	U=530.0 p=NS	U=621.0 p=NS
UbC/Gapdh	Mann Whitney test	U=590.0 p=NS	U=611.0 p=NS	U=316.0 p<0.001	U=346.0 p<0.001
Gapdh/b-Act	Student's t–test	t=0.9138 df=75 p=NS	t=0.4659 df=75 p=NS	t=0.4119 df=75 p=NS	t=0.8129 df=75 p=NS
β–Act/Tfrc	Mann Whitney test	U=461.0 p<0.05	U=556.0 p=NS	U=651.0 p=NS	U=559.0 p=NS
Tfrc	Mann Whitney test	U=436.0 p<0.01	U=633.0 p=NS	U=473.0 p<0.05	U=406.0 p<0.01

[a]NS: non–significant.
